# Analytical Methods for Causality Evaluation of Photonic Materials

**DOI:** 10.3390/ma15041536

**Published:** 2022-02-18

**Authors:** Tomasz P. Stefański, Jacek Gulgowski, Kosmas L. Tsakmakidis

**Affiliations:** 1 Faculty of Electronics, Telecommunications, and Informatics, Gdansk University of Technology, 80-233 Gdansk, Poland; 2 The Faculty of Mathematics, Physics and Informatics, University of Gdansk, 80-308 Gdansk, Poland; jacek.gulgowski@ug.edu.pl; 3 Section of Condensed Matter Physics, Department of Physics, National and Kapodistrian University of Athens, Panepistimioupolis, GR-157 84 Athens, Greece; ktsakmakidis@phys.uoa.gr

**Keywords:** photonic materials, causality, Kramers–Krönig relations, Titchmarsh theorem, Paley-Wiener theorem, distribution theory, fractional calculus

## Abstract

We comprehensively review several general methods and analytical tools used for causality evaluation of photonic materials. Our objective is to call to mind and then formulate, on a mathematically rigorous basis, a set of theorems which can answer the question whether a considered material model is causal or not. For this purpose, a set of various distributional theorems presented in literature is collected as the distributional version of the Titchmarsh theorem, allowing for evaluation of causality in complicated electromagnetic systems. Furthermore, we correct the existing material models with the use of distribution theory in order to obtain their causal formulations. In addition to the well-known Kramers–Krönig (K–K) relations, we overview four further methods which can be used to assess causality of given dispersion relations, when calculations of integrals involved in the K–K relations are challenging or even impossible. Depending on the given problem, optimal approaches allowing us to prove either the causality or lack thereof are pointed out. These methodologies should be useful for scientists and engineers analyzing causality problems in electrodynamics and optics, particularly with regard to photonic materials, when the involved mathematical distributions have to be invoked.

## 1. Introduction

The time-domain response of electromagnetic system has to be causal [1,2,3,4], i.e., a system cannot respond before the excitation starts. Hence, causality restricts the characteristics of any system, not only in the time domain but also in the frequency domain. Therefore, it is often stated in literature that real and imaginary parts of various complex parameters and characteristics (e.g., susceptibilities and frequency responses) are related by the Kramers–Krönig (K–K) integral relations [4]. The K–K relations are also valid between the modulus logarithm and the argument of the frequency-domain response (e.g., between the phase delay and the attenuation of a solution to a wave-propagation problem). However, it occurs that integrations in the K–K relations are not straightforward, and sometimes even impossible to perform. It stems from singular, highly oscillatory, or even diverging integrands, and infinite integration limits in the Hilbert transformation [5]. Therefore, the integrals cannot be evaluated in many important practical cases because they are divergent or undefined [6]. The problems concerning causality and the K–K relations are studied not only in electrodynamics [7,8,9,10,11] but also in acoustics [12,13,14,15,16], solid mechanics [17,18] and the control theory [19].

Although the subject of causality and dispersion of electromagnetic waves in photonic materials (e.g., dielectrics) was initiated in 1927 by the seminal papers of Kramers and Krönig [20,21], it has remained active in literature up to now [22]. Furthermore, classical books, devoted solely to this subject, are published [4]. As one can notice, although the book [4] was published in 1972, it has remained the initial point for many investigations referring to the subject, and constitutes the basic reference in this research area. On the other hand, a discussion related to origins of the Titchmarsh theorem recently appeared in literature [23]. This theorem establishes the equivalence between causality and the K–K relations. It occurs that it is a compilation of two important theorems, i.e., the Paley–Wiener theorem and the Marcel Riesz theorem. Therefore, the Authors of [23] have perceived the need for studying the subject with the use of rigorous mathematical tools. This approach, although often longer than the one presented in physics literature, has the advantage of being more precise.

Let us present some recent results related to causality in electrodynamics and optics. In [17], the K–K relations, which are valid for a general class of linear homogeneous or inhomogeneous media, are derived. That is, the proof of the K–K relations proceeds without *a priori* knowledge of wave velocity in the medium supporting the wave, when the frequency goes towards infinity. In [10], the K–K relations are derived for the effective index of modes propagating in optical waveguides. When material dispersion and absorption can be neglected within the frequency range of interest, the evanescent modes introduce an effective loss term in the K–K relations, meaning that these relations are valid even if material absorption is negligible within the frequency range of interest. In [11], a novel approach to the standard one is proposed for the derivation of the K–K relations for linear optical properties. That is, this approach is not based on contour integration and the Cauchy integral formula. Although this derivation still employs analytic behavior of the property under consideration, it employs only elementary properties of the Hilbert transformation to obtain the second formula of the K–K pair, from the Herglotz representation of the optical property as a Herglotz function. In [24], linear-response laws and causality within the time and frequency domains are analyzed in electrodynamics. It is demonstrated that one can violate causality in the frequency domain by making a vanishing-absorption approximation. Our contribution to this subject relies on generalization of the K–K relations for evaluation of causality in power-law media. In general, for square-integrable functions of the frequency, the validity of classical K–K relations is equivalent to causality in the time domain [1,4,17]. Things get complicated when the K–K relations are verified between the modulus logarithm and the argument. Then, the considered function does not belong to the class of square-integrable functions, and one can employ classical K–K relations with subtractions, but their satisfaction does not mean that the originally considered function is causal [13,17]. That is, the dispersion relation can be formulated for the considered system. It is based on the assumption that the subtracted logarithm of the response is causal, but the considered response function does not have to be causal. Therefore, we address this issue in [25], where the K–K relations are generalized towards non square-integrable functions. That is, the K–K relations with one and two subtractions are formulated. Their validity and satisfaction of additional assumptions imply causality of the considered function. Then, the formulated theory is applied to the analysis of electromagnetic media characterized by power-law frequency dispersion [25] and fractional-order (FO) models [26]. However, in our opinion, it might still be unclear for community members how to apply the developed mathematical tools to causality evaluation.

Therefore, we have decided to set in order the theory of causality and prepare the review of various analytical techniques allowing for evaluating causality of photonic materials. It includes the classical methods and models known from electrodynamics handbooks [7], as well as recently published results on causality evaluation [25,26]. Furthermore, we also consider various causality tests, which allow for establishing minimal limits for losses, for metamaterials [27,28]. Therefore, one can easily find their own approach to causality evaluation when a new model is formulated in classical electrodynamics. However, it is worth noticing that the procedure of canonical quantization of macroscopic electromagnetism [29] requires satisfaction of the K–K relations by dielectric functions of a linear, inhomogeneous, magnetodielectric medium. Hence, our review should hopefully also be useful in quantum-electrodynamics research. The paper begins with a short introduction of the notation, definitions, and basic mathematical theorems used throughout the paper. In Section 3, the problem of causality is presented, with various mathematical models applicable in electrodynamics and optics. However, we do not focus on physical motivations for these models, but we rather formulate mathematical approaches allowing for causality evaluation. Analytical methods for causality evaluation are presented in Section 4, whereas a set of examples is presented in Section 5. In this paper, the most important models of photonic materials are analyzed in terms of causality with the use of precise mathematical tools. Furthermore, the set of various distributional theorems presented in literature is collected as the distributional version of the Titchmarsh theorem, allowing us to evaluate causality of complicated electromagnetic systems on a mathematically rigorous basis. Hopefully, researchers interested in evaluating causality of novel models can use our paper as a template for their own mathematical derivations and proofs.

## 2. Review of Background Mathematics

### 2.1. Basic Notations

In this paper, a standard engineering notation is employed, which denotes an imaginary unit as $j=\sqrt{-1}$. For the complex number s=u+jv, we denote its real part as ℜs=u, and its imaginary part as ℑs=v. The (open) right half-plane is denoted as $C_{+}=\left\{s\in C:ℜs>0\right\}$. We denote the space of all functions which are holomorphic in the right half-plane and bounded by a polynomial as $H^{+}$. To be more precise: $G\in H^{+}$ means that the function *G* is holomorphic in the right half-plane, and there exist l∈N and a constant A(σ) such that for all σ>0

(1) $$\left|G\left(s\right)\right|\leq {A\left(\sigma \right)|s|}^{l}$$

for all $s\in C_{+}$ satisfying ℜs≥σ>0.

Then, we define the Fourier transformation for the absolutely integrable function f(t), using the formulation applied in electrical sciences

(2) $$F\left(f\right)\left(\omega \right)=F\left(\omega \right)=\int_{-\infty }^{+\infty }e^{-j\omega t}f\left(t\right)dt$$

whereas the inverse Fourier transformation is given by

(3) $$F^{-1}\left(F\right)\left(t\right)=f\left(t\right)=\frac{1}{2\pi }\int_{-\infty }^{+\infty }e^{j\omega t}F\left(\omega \right)d\omega .$$

In order to change the settings of the Fourier transformation to the one widely applied in mathematics and physics, one should replace the imaginary unit *j* with −i in (2) and (3) (where $i=\sqrt{-1}$) [8]. Various literature sources which we refer to sometimes apply the *mathematical i*-convention; hence we convert those results to the *engineering j*-convention.

Among various elementary functions, we often refer to the Heaviside step function u(t), defined as u(t)=1 for t≥0 and u(t)=0 for t<0. Then we refer to the *signum* function sgn(t) defined as sgn(t)=t/|t| for t≠0 and sgn(t)=0 for t=0. One should be aware that these functions appear as regular tempered distributions and, as such, should be identified up to the Lebesque measure zero sets. Hence, in both cases, it actually does not matter how the functions are defined for t=0.

In this paper, we often refer to the concept of Hölder continuous functions. We call the function f:R→C a Hölder continuous one (with an exponent α∈(0,1]), if there exists such a constant L>0 that

(4) $$|f\left(t_{1}\right)-f\left(t_{2}\right)\left|\leq L\right|t_{1}-t_{2}{|}^{\alpha }$$

for all $t_{1},t_{2}\in R$. If the condition (4) is satisfied on every interval [−M,M]⊂R (with a constant L=L(M) possibly depending on *M*), we can say that the function is *locally Hölder continuous*. We should mention here that the case α=1 corresponds to Lipschitz continuous functions. One can also notice that the class of locally Hölder continuous functions covers all the functions with a continuous derivative, but it is essentially larger (with the function $f\left(t\right)=\sqrt{\left|t\right|}$ as an example of Hölder continuous function with the exponent α=1/2).

In this paper, the Hilbert transformation [5] of the function f:R→R is applied. It is defined as

(5) $$\left(Hf\right)\left(x\right)=\frac{1}{\pi }{⨏}_{-\infty }^{+\infty }\frac{f(t)}{x-t}dt=\frac{1}{\pi }\lim_{\varepsilon \to 0^{+}}\int_{|x-t|\geq \varepsilon }\frac{f(t)}{x-t}dt.$$

The classical domain of this definition is the space $L^{q}\left(R\right)$ for any q∈(1,+∞). The definition (5) can be formulated for a wide class of distributions, which is discussed below.

### 2.2. Fractional Calculus

In modeling of dielectrics, one can find FO derivatives, hence the notation is fixed below. Various approaches to fractional calculus are considered in literature, i.e., Riemann-Liouville, Caputo, Grünwald–Letnikov and Marchaud, to mention just a few. For appropriate definitions, refer to classical monographs [30,31,32]. In this paper, when the fractional derivative of order α>0 is applied, its Marchaud definition is used (refer to [31] Sections 5.4 and 5.5)

(6) $$D^{\alpha }f\left(t\right)=\frac{\left\{\alpha \right\}}{\Gamma (1-\{\alpha \})}\int_{0}^{+\infty }\frac{f^{\left(n\right)}\left(t\right)-f^{\left(n\right)}\left(t-\tau \right)}{\tau^{1+\{\alpha \}}}d\tau$$

for α=n+{α} (n∈N∪{0} and {α}∈(0,1)). In (6), the function *f* is assumed to be sufficiently smooth, e.g., $f\in C^{n+1}\left(R\right)$ with $|f^{\left(n\right)}|$ bounded by a function which is not growing too quickly in ±∞.

The main reasons behind the usage of the Marchaud derivative in our considerations are as follows [33,34]:

- The Marchaud derivative of the order α∈(0,1) for the function *f* exists, if f:R→R is bounded and locally Hölder with an appropriate exponent.
- For the Marchaud derivative of the order α of the exponential function $e^{j\omega t}$ (where ω∈R is fixed), one obtains
  (7) $$D^{\alpha }e^{j\omega t}={\left(j\omega \right)}^{\alpha }e^{j\omega t}.$$
- The Marchaud and Grünwald–Letnikov derivatives coincide for a very broad class of functions.
- The Marchaud derivative satisfies the semigroup property for all the *f* functions for which this definition coincides with the Grünwald–Letnikov definition, i.e., $D^{\alpha }\left(D^{\beta }f\right)=D^{\alpha +\beta }\left(f\right)$ where α,β>0.

It is demonstrated in [33,34] that, in order to obtain the equivalence between the results in the time and frequency domains, the FO derivative modeling electromagnetic systems should be representable in the phasor domain (i.e., satisfy (7)) and satisfy the semigroup property. From this point of view, we considered in [33,34] the following definitions of FO derivatives applied for the electromagnetic modeling: Riemann–Liouville, Caputo, Liouville–Caputo, Liouville, Marchaud, Grünwald–Letnikov, Caputo–Fabrizio, Atangana–Baleanu, Atangana–Koca–Caputo and the conformable derivative. Out of these most popular approaches, only the Grünwald–Letnikov and Marchaud definitions (which are actually equivalent for a wide class of functions) satisfy the semigroup property and are naturally representable in the phasor domain. Therefore, we employ the Marchaud derivative in our research focusing on causality evaluation of photonic materials.

### 2.3. Distribution Theory

The distribution theory is applied in our investigations; therefore the notation is fixed below. It is a very formal mathematical theory, which is widely applied in many branches of science and engineering. However, the very formal formulation of this theory, e.g., proposed in purely mathematical books [35,36], is not straightforwardly applicable in applied physics and engineering. Even though alternative attitudes allow for different views on distributions, they are based on the same foundations. Therefore, we refer a reader to literature sources [37,38,39], which provide different perspectives on the distribution theory.

The support of the continuous function φ:R→R is the set [37]

(8) $$\mathrm{supp}\left(\varphi \right)=\bar{\left\{t\in R:\varphi (t)\neq 0\right\}}.$$

From now on, D denotes the space of *test functions* of the class $C^{\infty }\left(R\right)$ with compact support, endowed with appropriate topology (i.e., with the formal definition of all convergent sequences within the space of test functions). The topology is given by an appropriate family of seminorms, as described in Section 1.2.6 of [38]. The space dual to D, i.e., the space of *distributions*, is denoted as $D^{\prime }$. The linear continuous functional *f* on D is denoted using the *dual-pair* notation 〈f,φ〉, where $f\in D^{\prime }$ and φ∈D. The space of Schwartz functions, i.e., *rapidly decreasing functions*, is denoted as S. Its dual space, i.e., the space of *tempered distributions*, is denoted as $S^{\prime }$. The Fourier transformation is defined for all tempered distributions by the formula [38] Section 3.1.4

(9) 〈F(f),φ〉=〈f,F(φ)〉

for all φ∈S. The support of the distribution *f* is defined as the set supp(f) being a complement of the largest open set *U*, on which *f* vanishes [38] Section 1.3.1

(10) $$\forall_{\varphi \in D}\;\mathrm{supp}\left(\varphi \right)\subset U\Rightarrow \langle f,\varphi \rangle =0.$$

Let E denote the space of all $C^{\infty }\left(R\right)$ functions. Then its dual $E^{\prime }$ is the space of *distributions with compact support*.

We should also refer to some spaces of test functions (and related spaces of distributions), which are useful when discussing the Hilbert transformation. Let us take q∈(1,+∞) and $q^{\prime }$ such that $1/q+1/q^{\prime }=1$. Then, the space of test functions $D_{L^{q^{\prime }}}\subset C^{\infty }\left(R\right)$ consists of all $\varphi \in C^{\infty }\left(R\right)$ such that all the derivatives $\varphi^{\left(k\right)}\in L^{q^{\prime }}\left(R\right)$ for all k=0,1,2… with the topology defined by the family of seminorms inherited from Sobolev spaces (for details, one is referred to (1.57) in [35]). Let $D_{L^{q}}^{\prime }$ denote the space dual to $D_{L^{q^{\prime }}}$. The special case is q=2 with $D_{L^{2}}^{\prime }$ being dual to $D_{L^{2}}$. The derivative of the order *k* of the function (or distribution) *f* is denoted as $D^{k}f$. For the space $D_{L^{q}}^{\prime }$, one can formulate the following theorem:

**Theorem** **1** ([35] Theorem 1.26)**.** *The distribution u belongs to $D_{L^{q}}^{\prime }$ iff there exists such a positive integer m and such $L^{q}\left(R\right)$ functions $u_{0},u_{1},\ldots ,u_{m}$ that $u=\sum_{l=0}^{m}D^{l}u_{l}$ (where the derivatives are in the distributional sense).*

One can find a detailed discussion of properties of the spaces $D_{L^{q}}^{\prime }$ in Section 10.2 in [5].

The relation between different subspaces of the space of distribution $D^{\prime }$ can be summarized as (see [5] Section 10.2)

(11) $$E^{\prime }\subset D_{L^{q}}^{\prime }\subset D_{L^{r}}^{\prime }\subset S^{\prime }\subset D^{\prime }$$

where q≤r.

Let us define the distribution p.v.(1/x), which is needed in the sequel, as follows:

(12) $$\langle p.v.(1/x),\varphi \rangle ={⨏}_{-\infty }^{\infty }\frac{\varphi (y)}{y}dy=\lim_{\varepsilon \to 0^{+}}\int_{|y|\geq \varepsilon }\frac{\varphi (y)}{y}dy.$$

As shown in [5] Section 10.7, the distribution p.v.(1/x) belongs to $D_{L^{q}}^{\prime }$ for all q∈(1,+∞).

In [35] Lemma 1.8, the space $S_{0}$ is defined as the set of all tempered distributions such that $F\left(S_{0}\right)=D_{L^{2}}^{\prime }$. Following this definition, $S_{q}$ is defined as the space of such tempered distributions that $F\left(S_{q}\right)=D_{L^{q}}^{\prime }$, and the Fourier transformation is a one-to-one mapping between $S_{q}$ and $D_{L^{q}}^{\prime }$.

If one considers two distributions $f,g\in D^{\prime }$, then its convolution is not always well-defined (refer to the discussion in Section 10.6 of King’s book [5]). However, when it is possible to define the convolution of distributions *f* and *g*, it is defined as the distribution *h* such that

(13) $$\langle h,\varphi \rangle =\langle f(x),\langle g(y),\varphi (x+y)\rangle \rangle$$

for the test function φ∈D. There are some cases, when the convolution of distributions is well-defined (refer to the end of Sections 10.6 and 10.7 in [5]), that is:

- if $f\in D^{\prime }$ and $g\in E^{\prime }$, then $f*g\in D^{\prime }$
- if $f\in S^{\prime }$ and $g\in E^{\prime }$, then $f*g\in S^{\prime }$
- if $f\in E^{\prime }$ and $g\in E^{\prime }$, then $f*g\in E^{\prime }$
- if $f,g\in D_{L^{2}}^{\prime }$, then f∗g exists
- if $f\in D_{L^{p}}^{\prime }$, $g\in D_{L^{q}}^{\prime }$ and 1/p+1/q≥1 then f∗g exists, and $f*g\in D_{L^{r}}^{\prime }$, where 1/r=1/p+1/q−1.

The Hilbert transformation of the distribution $f\in D_{L^{q}}^{\prime }$ is defined as the distribution H(f) (see [5] Equation (10.83)) satisfying the formula

(14) 〈H(f),φ〉=−〈f,H(φ)〉

where $\varphi \in D_{L^{q^{\prime }}}$. For the function $f\in L^{q}\left(R\right)$, the Hilbert transformation can be written as the convolution

(15) $$H\left(f\right)=\frac{1}{\pi }f*p.v.\left(1/x\right),$$

which is defined in the distributional sense (see [5] Equation (10.102) and the following discussion ending at Equation (10.121)).

We should bear in mind some important properties of the distributional Hilbert transformation. That is, the following properties are valid for q∈(1,+∞):

- For any distribution $f\in D_{L^{q}}^{\prime }$, one obtains (cf. Section 10.9 in [5])
  (16) H(H(f))=−f.
- Let $f\in L^{q}\left(R\right)$ be a function. Then one obtains (see (4.30) in [5])
  (17) H(f(ax+b))(y)=sgn(a)H(f(x))(ay+b).
- For any $f\in D_{L^{q}}^{\prime }$, the Hilbert transformation commutes with the distributional derivative (see (10.173) and the entire Section 10.10 in [5])
  (18) DH(f)=H(Df).
- The Hilbert transformation of the Dirac delta is given by (see (10.85) in [5])
  (19) $$H\left(\delta \left(x\right)\right)\left(y\right)=\frac{1}{\pi }p.v.\left(1/y\right).$$
- The distribution p.v.(1/x) belongs to $D_{L^{q}}^{\prime }$, and one obtains (see (10.87) in [5])
  (20) $$H\left(p.v.(1/x)\right)\left(y\right)=-\pi \delta \left(y\right).$$
- A similar result to the one given above can be stated for the translated distribution $f=\;p.v.\frac{1}{x-a}$. That is, one obtains
  (21) $$H\left(p.v.\frac{1}{x-a}\right)\left(y\right)=-\pi \delta \left(y-a\right).$$
  Although the above property is quite obvious, we were not able to find it given directly in literature. Therefore, a short proof of this property is given. Let us take the test function $\varphi \in D_{L^{q^{\prime }}}$ and check (we use the definition (14), the variable change property (17) for the transformation of $L^{q}\left(R\right)$ function, and additionally the change of variables x−a↦x)
  $$\langle Hp.v.\frac{1}{x-a},\varphi \rangle =-\langle p.v.\frac{1}{x-a},H\varphi \rangle =-\lim_{\varepsilon \to 0^{+}}\int_{|x-a|\geq \varepsilon }\frac{1}{x-a}\left(H\left(\varphi \right)\right)\left(x\right)dx=$$
  $$-\lim_{\varepsilon \to 0^{+}}\int_{|x|\geq \varepsilon }\frac{1}{x}\left(H\left(\varphi \right)\right)\left(x+a\right)dx=-\lim_{\varepsilon \to 0^{+}}\int_{|x|\geq \varepsilon }\frac{1}{x}\left(H\left(\varphi \left(\cdot +a\right)\right)\right)\left(x\right)dx=$$
  $$\langle H\left(p.v.\frac{1}{x}\right),\varphi \left(\cdot +a\right)\rangle =-\pi \langle \delta ,\varphi \left(\cdot +a\right)\rangle =-\pi \varphi \left(a\right)$$
  which completes the proof of (21).

We also need to remember topology in the space $S^{\prime }$ of tempered distributions: One says that the sequence of tempered distributions $\left(f_{n}\right)\subset S^{\prime }$ converges to a tempered distribution $f\in S^{\prime }$ if

(22) $$\langle f_{n},\varphi \rangle \to \langle f,\varphi \rangle$$

for any Schwartz function φ∈S [37].

## 3. Causality in Electrodynamics and Optics

Let us consider Maxwell’s equations in the time domain

(23) ∇·D=ρ

(24) $$\nabla \times E=-\frac{\partial B}{\partial t}$$

(25) ∇·B=0

(26) $$\nabla \times H=\frac{\partial D}{\partial t}+J$$

where E and H denote, respectively, the electric- and magnetic-field intensities, D and B denote, respectively, the electric- and magnetic-flux densities, J and ρ denote, respectively, the current and charge densities. In this paper, we focus on dielectric properties of isotropic electromagnetic media, i.e., photonic materials, whose properties are described in the frequency domain. It stems from the fact that the number of models of dielectric properties of media is much larger than for magnetic properties. For such mathematical models, it is crucial to approximate physical characteristics by using causal formulas. However, the presented methods and tools can be extended towards magnetic characteristics. Furthermore, the obtained solutions of Maxwell’s equations should also be causal. Finally, having the frequency response of media in the wave-propagation (or wave-guiding) problem, one can evaluate causality using the theorems presented below, supported by methods of their usage.

### 3.1. Basic Definitions

The function f:R→R (generally f:R→C) or the distribution $f\in D^{\prime }$ is called *causal* if its support supp(f)⊂[0,+∞). The Fourier transform F=F(f) is called a *causal transform* if $\mathrm{supp}(F^{-1}\left(F\right))\subset \left[0,+\infty \right)$. In other words, one can assume that f(t) is causal if it can be represented as f(t)=u(t)g(t), where u(t) is the Heaviside step function and g(t)=f(t) for t>0 [40]. In practical terms, one should also assume that g(t) is the function whose Laplace transform has a non-degenerate region of convergence.

In terms of physics, causality means that the effect does not precede the cause. Hence, if one considers the electromagnetic system whose time-domain function f(t) describes the system response to the Dirac delta excitation, then causality means that f(t)=0 for t<0. It also means that response of the system depends only on excitation values from the past. To sum up, the mathematical definitions of causality formulated above closely follow physical understanding of this term.

### 3.2. Dielectric Models

Transformation of (23)–(26) into the frequency domain gives

(27) $$\nabla \cdot \tilde{D}=\tilde{\rho }$$

(28) $$\nabla \times \tilde{E}=-j\omega \tilde{B}$$

(29) $$\nabla \cdot \tilde{B}=0$$

(30) $$\nabla \times \tilde{H}=j\omega \tilde{D}+\tilde{J}$$

where tilde denotes phasor representation of the physical quantity, i.e., $a\left(t\right)=ℜ\left(\tilde{a}e^{j\omega t}\right)$, and ω denotes the angular frequency. To solve this set of equations, one additionally needs constitutive relations between $\tilde{D}$ and $\tilde{E}$ as well as between $\tilde{B}$ and $\tilde{H}$. Hence, one can write

(31) $$\tilde{D}=\epsilon \left(\omega \right)\tilde{E}$$

(32) $$\tilde{B}=\mu \left(\omega \right)\tilde{H}.$$

Then one can also write that

(33) $$\tilde{D}=\epsilon_{0}\tilde{E}+\tilde{P}=\epsilon_{0}\left(1+\chi_{e}\left(\omega \right)\right)\tilde{E}$$

(34) $$\tilde{B}=\mu_{0}\tilde{H}+\tilde{M}=\mu_{0}\left(1+\chi_{m}\left(\omega \right)\right)\tilde{H}$$

where $\tilde{P}=\epsilon_{0}\chi_{e}\left(\omega \right)\tilde{E}$ and $\tilde{M}=\mu_{0}\chi_{m}\left(\omega \right)\tilde{H}$ are, respectively, the electric and magnetic polarizations, $\epsilon_{0}$ and $\mu_{0}$ are, respectively, the vacuum permittivity and permeability, $\chi_{e}\left(\omega \right)$ and $\chi_{m}\left(\omega \right)$ are, respectively, the electric and magnetic susceptibilities of the medium. As it has already been mentioned, our considerations are focused on isotropic dielectric media. Therefore, below we consider general χ(ω) functions, which are mainly related to dielectrics, but can also represent, in an obvious way, magnetic models of media (i.e., magnetic susceptibility).

In electrodynamics, it is required that χ(ω) is a causal transform [7], because all dielectrics are not able to polarize instantaneously in response to an applied field. Alternatively, one can require that the function $\frac{\epsilon (\omega )}{\epsilon_{0}}-1$ is a causal transform.

Let us formulate the first considered dielectric model, in which the transform ϵ(ω) is a real constant. That is

(35) $$\epsilon \left(\omega \right)=\epsilon =\epsilon_{0}\epsilon_{r}$$

where $\epsilon_{r}$ is the constant relative permittivity. Then let us assume the ohmic conduction, i.e., the current flow whose density depends on the electric-field intensity as follows:

(36) $$\tilde{J}=\sigma \tilde{E}.$$

In (36), σ∈R denotes the electrical conductivity. Therefore, assuming that ω≠0, (30) can be written as

(37) $$\nabla \times \tilde{H}=j\omega \epsilon \left(\omega \right)\tilde{E}$$

where

(38) $$\epsilon \left(\omega \right)=\epsilon_{0}\epsilon_{r}-j\frac{\sigma }{\omega }$$

includes ohmic losses within the complex permittivity ϵ(ω). This result can be generalized towards

(39) $$\epsilon \left(\omega \right)=\epsilon^{\prime }\left(\omega \right)-j\epsilon^{\prime \prime }\left(\omega \right)$$

where $\epsilon^{\prime }\left(\omega \right),\epsilon^{\prime \prime }\left(\omega \right)\in R$ are functions of frequency and $\epsilon^{\prime \prime }\left(\omega \right)$ describes all losses in the electric field. If the losses in a dielectric material stem from ohmic conduction, then $\epsilon^{\prime \prime }\left(\omega \right)$ is proportional to 1/ω. Otherwise, if losses stem from the bound charge and dipole relaxation phenomena, then $\epsilon^{\prime \prime }\left(\omega \right)$ is not proportional to 1/ω. Analogously, one can write for the permeability

(40) $$\mu \left(\omega \right)=\mu^{\prime }\left(\omega \right)-j\mu^{\prime \prime }\left(\omega \right)$$

where $\mu^{\prime }\left(\omega \right),\mu^{\prime \prime }\left(\omega \right)\in R$ are functions of frequency.

Let us formulate a few popular models describing the response of dielectrics with the use of permittivity:

- Djordjevic-Sarkar relationship for lossy dielectrics [6,41]
  (41) $$\epsilon \left(\omega \right)=\epsilon_{0}\left(1+\frac{\Delta \epsilon_{r}^{\prime }}{log_{10}\frac{\omega_{2}}{\omega_{1}}}\frac{ln\frac{\omega_{2}+j\omega }{\omega_{1}+j\omega }}{ln10}-j\frac{\sigma }{\omega \epsilon_{0}}\right)$$
  where ω≠0. The first term in (41) is the relative permittivity at very high frequencies, the second term is the broadband logarithmic term, and the third term comes from conductivity. In (41), $\Delta \epsilon_{r}^{\prime }$, $\omega_{1}$, $\omega_{2}$ are model parameters. This formula gives a simple closed-form expression, which approximates the measured permittivity of the popular FR-4 substrate used for manufacturing printed circuit boards.
- Westerlund relationship for FO capacitors [42,43]
  (42) $$\epsilon \left(\omega \right)=\frac{\epsilon_{\beta }}{{\left(j\omega \right)}^{1-\beta }},\beta \in \left[0,1\right]$$
  where ω≠0. It allows for formulating the constitutive relation (31) in the time domain with the use of FO derivative as
  (43) $$\epsilon_{\beta }E=D_{t}^{1-\beta }D,\beta \in \left[0,1\right].$$
  Although this model does not explain the nature of internal processes in dielectrics, it reproduces and predicts their behavior much better than any other theory (according to the Authors of [42]). Therefore, it is referred to as an ‘engineering’ model of dielectrics. Furthermore, this model allows for obtaining the electrical characteristics of FO capacitors, (refer to [43,44]).
- Power-law relationship for porous media [45]
  (44) $$\epsilon \left(\omega \right)=A\omega^{-\alpha },\alpha \in \left[0,1\right]$$
  where ω≠0, ϵ(ω)∈R, *A* is a constant, and α is close to 1.0 in a low frequency region, and is within the range of 0–0.5 in a high frequency region. The model (44) describes the permittivity of porous media such as wet soils and sedimentary rocks, which has been observed to be considerably different than in the case of water and parent minerals.

### 3.3. Dielectric Relaxation

Let us formulate several popular dielectric models based on electric susceptibility χ(ω) in the complex domain (τ>0 is the relaxation time, and $\Delta \epsilon_{r}=\frac{\epsilon_{s}-\epsilon_{0}}{\epsilon_{0}}$ where $\epsilon_{s}=\epsilon \left(\omega \to 0^{+}\right)$):

- Debye [46,47]
  (45) $$\chi \left(\omega \right)=\frac{\Delta \epsilon_{r}}{1+j\omega \tau }.$$
  This model is frequently used to describe simple dielectric characteristics of electromagnetic media arising from bipolar relaxation. The Formula (45) is characterized by a single relaxation time, which is capable of handling materials with high-water content. However, experimental studies show that the relaxation behavior of a wide range of dielectrics strongly differs from the Debye relaxation formula. Therefore, a number of phenomena such as broadness, asymmetry and excess in dielectric dispersion has motivated the development of empirical response functions described below, such as Cole-Cole, Cole-Davidson, Havriliak-Negami, and Raicu [46].
- Lorentz [7,47]
  (46) $$\chi \left(\omega \right)=\frac{\Delta \epsilon_{r}\omega_{0}^{2}}{\omega_{0}^{2}+2j\omega \gamma -\omega^{2}}$$
  where $\omega_{0}$ is the frequency of a pole pair (the undamped resonant frequency of the medium), and γ is the damping coefficient. The model is based on the classical theory of light-matter interaction, and describes the frequency-dependant polarization due to bound charges. That is, bindings between electrons and nucleus in atom are treated similarly to those of the mass-spring harmonic-oscillator system. It is worth mentioning that any function obeying the K–K relations can be approximated as a superposition of Lorentzian functions, to any precision [48]. Therefore, the Lorentzian function (46) can be considered as a general building block for implementing causal susceptibilities of various materials, e.g., metamaterials.
- Lorentz in high-frequency limit [7]
  (47) $$\chi \left(\omega \right)=-\frac{\omega_{p}^{2}}{\omega^{2}}$$
  where ω≠0 and $\omega_{p}$ is the plasma frequency of medium. This model results from (46), assuming that the frequency is far above the highest resonant frequency $\omega_{0}$ in the medium.
- Lorentz in high-frequency limit with static magnetic induction [7]
  (48) $$\chi \left(\omega \right)=-\frac{\omega_{p}^{2}}{\omega (\omega \pm \omega_{B})}$$
  where ω≠0 and $\omega \neq \mp \omega_{B}$. In (48), $\omega_{B}$ is the frequency of precession of a charged particle in magnetic field. This model is the extension of (47), which involves an interaction between static magnetic field $B_{0}$ and tenuous electronic plasma of uniform density, when transverse waves propagate parallel to the direction of $B_{0}$.
- Lorentz in FO generalization [49]
  (49) $$\chi \left(\omega \right)=\frac{{\left(\frac{\omega_{p}}{\omega_{0}}\right)}^{2}}{1+2\gamma {\left(j\frac{\omega }{\omega_{0}}\right)}^{\alpha }+{\left(j\frac{\omega }{\omega_{0}}\right)}^{2\alpha }},\alpha \in \left(0,1\right]$$
  where $\omega_{p}$ is the termed bulk plasma frequency associated with electrons, and γ is the damping coefficient. This model extends the classical Lorentz model (46) for a dielectric material with the use of FO derivatives, but it is formulated in the frequency domain.
- Drude [47]
  (50) $$\chi \left(\omega \right)=-\frac{\omega_{0}^{2}}{\omega^{2}-j\omega \gamma }$$
  where ω≠0 and ω≠jγ. In (50), $\omega_{0}$ is the Drude pole frequency and γ is the inverse of the pole relaxation time. This model results from the application of kinetic theory to electrons in solids for optical frequencies. It can be obtained from the aforementioned Lorenz model (i.e., harmonic-oscillator model) when the restoration force is removed (i.e., free electrons are assumed which are not bound to a particular nucleus).
- Cole-Cole [46,50,51,52]
  (51) $$\chi \left(\omega \right)=\frac{\Delta \epsilon_{r}}{1+{\left(j\omega \tau \right)}^{\alpha }},\alpha \in \left[0,2\right].$$
  This model has been developed as an empirical extension of the Debye model (45), which can be obtained for α=1.
- Cole-Davidson [46,53]
  (52) $$\chi \left(\omega \right)=\frac{\Delta \epsilon_{r}}{{\left(1+j\omega \tau \right)}^{\beta }},\beta \in \left[0,1\right].$$
  This model has been developed as an empirical extension of the Debye model (45), which can be obtained for β=1.
- Havriliak-Negami [46,54,55]
  (53) $$\chi \left(\omega \right)=\frac{\Delta \epsilon_{r}}{{\left(1+{\left(j\omega \tau \right)}^{\alpha }\right)}^{\beta }},\alpha ,\beta \in \left[0,1\right].$$
  This model extends Cole-Cole (51) and Cole-Davidson (52) models.
- Raicu [46,56]
  (54) $$\chi \left(\omega \right)=\frac{\Delta }{{\left({\left(j\omega \tau \right)}^{\gamma }+{\left(j\omega \tau \right)}^{\alpha }\right)}^{\beta }},\alpha ,\beta ,\gamma \in \left[0,1\right]$$
  where Δ is the relative dielectric increment in the Raicu model. This model extends (53) by including the additional parameter γ.
- Universal dielectric response [57,58,59]
  (55) $$\chi \left(\omega \right)=\chi_{\alpha }{\left(j\omega \right)}^{-\alpha },\alpha \in \left(0,1\right)$$
  where ω≠0 and $\chi_{\alpha }$ is a positive constant. In general, this model is valid for $\omega >>\omega_{p}$, where $\omega_{p}$ is the loss-peak frequency. It describes the observed behavior of dielectric properties demonstrated by solid-state systems. That is, it involves power-law scaling of dielectric properties with frequency, which is widely observed in nature.

A particular dielectric model can be a weighted sum of the several susceptibility characteristics χ(ω) presented above. For instance, the characteristic can be a weighted sum of several Lorentz functions (46), defined for different pole pairs and damping coefficients [7]. In such a case, if each of the components is causal, then the considered model is causal as well.

### 3.4. Frequency Response

If one obtains a causal solution to Maxwell’s Equations (23)–(26), then each of the components of the vector field E, D, H, B can depend only on the previous excitation values ρ, J. For instance, the relation between the excitation J and the response of electromagnetic system (E, H) in the frequency domain can be written as follows [8,60]:

(56) $$\tilde{E}\left(r\right)=\int_{V}G_{\mathrm{ee}}\left(r,r^{\prime }\right)\cdot \tilde{J}\left(r^{\prime }\right)\;dv^{\prime }$$

(57) $$\tilde{H}\left(r\right)=\int_{V}G_{\mathrm{me}}\left(r,r^{\prime }\right)\cdot \tilde{J}\left(r^{\prime }\right)\;dv^{\prime }.$$

In (56) and (57), $G_{\mathrm{ee}}\left(r,r^{\prime }\right)$, $G_{\mathrm{me}}\left(r,r^{\prime }\right)$ denote dyadic Green’s functions of electric-electric and magnetic-electric type, respectively. Because the considered electromagnetic-field systems are linear and time-invariant, it is possible to write the relation between a single excitation component (X(ω)) and a single output component (Y(ω)) in the frequency domain. Then, one obtains

(58) Y(ω)=G(ω)X(ω)

where G(ω) denotes frequency response of the system.

For instance, let us consider one-dimensional (1-D) propagation of a monochromatic plane wave along the *z* direction in a medium described by material parameters ϵ(ω) and μ(ω). Then one can write the following Helmholtz equation for $\tilde{E}=\tilde{E}\left(z\right)i_{x}$ and $\tilde{H}=\tilde{H}\left(z\right)i_{y}$:

(59) $$\nabla^{2}\tilde{E}\left(z\right)+k^{2}\tilde{E}\left(z\right)=\nabla \frac{\tilde{\rho }\left(z\right)}{\epsilon (\omega )}+j\omega \mu \left(\omega \right)\tilde{J}\left(z\right).$$

In (59), $k^{2}=\omega^{2}\mu \left(\omega \right)\epsilon \left(\omega \right)$ is the square of complex-valued wavenumber. In optics, the refractive index n(ω) is mainly used to describe an electromagnetic medium, which is a dimensionless number describing how fast the light travels through the medium. That is, n(ω)=c/v(ω), where v(ω) is the velocity of light in the medium and *c* is the velocity of light in the vacuum. Hence, one can also write that $k^{2}=n^{2}\left(\omega \right){\left(\omega /c\right)}^{2}$. If the refractive index is a complex number for a given angular frequency, its real part indicates the phase velocity, whereas its imaginary part describes the attenuation of electromagnetic waves in the medium. Let us consider the signalling problem [61,62,63], where the electric field in the homogeneous Equation (59) is excited by a source at the spatial-domain boundary. Hence one obtains

(60) $$\nabla^{2}\tilde{E}\left(z\right)+k^{2}\tilde{E}\left(z\right)=0$$

whose general solution is given by

(61) $$\tilde{E}\left(z\right)={\tilde{E}}_{+}e^{-jkz}+{\tilde{E}}_{-}e^{+jkz}.$$

in (61), ${\tilde{E}}_{+}$ and ${\tilde{E}}_{-}$ denote, respectively, complex amplitudes of waves propagating in the +z and −z directions, and +k and −k are complex roots of $k^{2}$. Considering wave propagation (or guiding) in the +z direction only, the propagation constant jk depends on the choice of ϵ(ω) and μ(ω) functions, and is selected as the one with a positive real part. Hence one obtains

(62) $$\tilde{E}\left(z\right)={\tilde{E}}_{+}e^{-jkz}$$

where ${\tilde{E}}_{+}$ is equal to $\tilde{E}\left(z=0\right)$. Such a solution is physically equivalent to impinging of the plane wave on the half-space constituting a medium described by ϵ(ω) and μ(ω). Then the wave is transferred into the medium and its time-domain waveform can be obtained as described in [25]. Alternatively, one can consider (62) as a general solution of the wave-guiding problem for, e.g., optical waveguide. Assuming the fixed length of the wave-propagation distance z=L, and taking $X\left(\omega \right)=\tilde{E}\left(z=0\right)$ and $Y\left(\omega \right)=\tilde{E}\left(z=L\right)$, the Formula (62) can be considered as the relation (58) where

(63) $$G\left(\omega \right)=e^{-jkL}.$$

Such a function is usually required to be a causal transform in electrodynamics. Furthermore, one can require that G(ω) is relativistically causal. That is, not only the inverse Fourier transform of G(ω) is equal to zero for t<0, but the inverse Fourier transform of $G\left(\omega \right)e^{j\omega L/c}$ is also equal to zero for t<0.

Let us consider FO models in electrodynamics, which start with constitutive relations as follows [43,44,63]:

(64) $$J=\sigma_{\alpha }D_{t}^{1-\alpha }E,\;0<\alpha \leq 1$$

(65) $$\epsilon_{\beta }E=D_{t}^{1-\beta }D,\;0<\beta \leq 1$$

(66) $$\mu_{\gamma }H=D_{t}^{1-\gamma }B,\;0<\gamma \leq 1.$$

Equation (43) is repeated here as (65) for the sake of completeness. When these relations are applied to Maxwell’s Equations (23)–(26), then one obtains the FO Maxwell’s equations in the following form:

(67) ∇·E=0

(68) $$\nabla \times E=-\mu_{\gamma }D_{t}^{\gamma }H$$

(69) ∇·H=0

(70) $$\nabla \times H=\epsilon_{\beta }D_{t}^{\beta }E+\sigma_{\alpha }D_{t}^{1-\alpha }E.$$

In order to analyze the 1-D propagation of a monochromatic plane wave along the *z*-direction, one can apply the phasor-domain representation and arrive at the special version of the Helmholtz Equation (59) with

(71) $$k^{2}\left(\omega \right)=-\left(\mu_{\gamma }\epsilon_{\beta }{\left(j\omega \right)}^{\beta +\gamma }+\sigma_{\alpha }\mu_{\gamma }{\left(j\omega \right)}^{1-\alpha +\gamma }\right).$$

With the additional assumption of the lack of current and charge sources within the considered space, and assuming that there is no power dissipation due to Joule’s heating, i.e., the current density is related to the electric-field intensity by the classical Ohm law (α=1)

(72) $$J=\sigma_{1}E$$

with the conductivity $\sigma_{1}=0$, the frequency response (i.e., the transfer function in the frequency domain) is given by (cf. [63])

(73) $$G\left(\omega \right)=e^{-z\sqrt{\mu_{\gamma }\epsilon_{\beta }}{\left(j\omega \right)}^{\frac{\beta +\gamma }{2}}}=e^{-z\sqrt{\mu_{\gamma }\epsilon_{\beta }}{\left(j\omega \right)}^{\nu }}$$

where $\nu =\frac{\beta +\gamma }{2}$.

For α=1, $\sigma_{1}=0$ and β=γ, one can write (67)–(70) in a compact form

(74) $$\nabla \times F=j\sqrt{\mu_{\beta }\epsilon_{\beta }}D_{t}^{\beta }F$$

(75) ∇·F=0

where

(76) $$F=\frac{1}{\sqrt{2}}\left(\frac{E}{\sqrt{Z_{f}}}+jH\sqrt{Z_{f}}\right)$$

is the Riemann-Silberstein (RS) vector in the time-fractional electrodynamics [64] and $Z_{f}=\sqrt{\frac{\mu_{\beta }}{\epsilon_{\beta }}}$. Then, one can write the diffusion-wave equation in time domain for space without sources

(77) $$\nabla^{2}F-\mu_{\beta }\epsilon_{\beta }D_{t}^{2\beta }F=0.$$

Assuming the plane-wave, spherical and cylindrical symmetries of solutions to (77), one obtains, respectively, the following transfer functions describing the 1-D wave propagation [64]:

(78) $$G_{z}\left(\omega \right)=e^{-z\sqrt{\mu_{\beta }\epsilon_{\beta }}{\left(j\omega \right)}^{\beta }}$$

(79) $$G_{R}\left(\omega \right)=\frac{1}{4\pi }\frac{1}{R}e^{-R\sqrt{\mu_{\beta }\epsilon_{\beta }}{\left(j\omega \right)}^{\beta }},R>0$$

(80) $$G_{r}\left(\omega \right)=-\frac{j}{4}J_{0}\left(j{\left(j\omega \right)}^{\beta }r\sqrt{\mu_{\beta }\epsilon_{\beta }}\right),r>0.$$

In (80), the function $J_{0}$ is the Bessel function of the first kind of zero order, i.e.,

(81) $$J_{0}\left(z\right)=\sum_{k=0}^{+\infty }{\left(-1\right)}^{k}\frac{1}{\Gamma {\left(k+1\right)}^{2}}{\left(\frac{z}{2}\right)}^{2k}.$$

## 4. Methods and Analytical Tools for Causality Evaluation

Let us consider a complex-valued transfer function G:R→C or distribution, which is the Fourier transform of a certain time-domain function g:R→C or distribution. It is worth noticing that we do not assume *a priori* that g(t) is real-valued.

**Figure 1:**
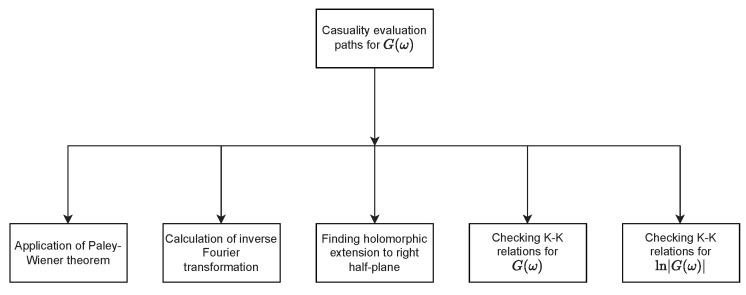
Approaches to causality evaluation.

Approaches to causality evaluation are presented in Figure 1. Having G(ω), one can evaluate causality by way of applying the Paley–Wiener theorem, calculating the inverse Fourier transformation, finding a holomorphic extension to the right half-plane, or checking various forms of the K–K relations. One should notice that, for the considered function G(ω), not every approach can easily be applied to prove either causality or lack thereof.

### 4.1. Paley–Wiener Theorem

Let us start our considerations from the Paley–Wiener theorem, which allows for characterisation of the *modulus* of the complex-valued $L^{2}$ function in terms of causality.

**Theorem** **2** (Paley–Wiener, [65] Theorem XII)**.** *Let ϕ(ω) be a real nonnegative function, not equivalent to 0 and belonging to $L^{2}\left(R\right)$. A necessary and sufficient condition that there should exist a real- or complex-valued function g(t), vanishing for $t\leq t_{0}$, for some number $t_{0}$, and such that the Fourier transform G(ω)=F(g(t))(ω) should satisfy |G(ω)|=ϕ(ω), is that*

(82) $$\int_{-\infty }^{+\infty }\frac{\left|\ln(\phi (\omega ))\right|}{1+\omega^{2}}d\omega <+\infty .$$

One should notice that the Paley–Wiener theorem does not state that the complex-valued function G(ω) is a causal transform. It states that, for the modulus ϕ(ω) satisfying (82), the causal transform G(ω) exists with the same modulus. It also states that if ϕ(ω)=|G(ω)| does not satisfy (82), then G(ω) is surely not a causal transform. This theorem is a valuable tool, which can be used to prove that the transfer function G(ω) is not a causal transform.

### 4.2. Calculation of Inverse Fourier Transformation

The simplest approach to causality evaluation relies on calculating the inverse Fourier transformation of the function G(ω). Then $g\left(t\right)=F^{-1}\left(G\left(\omega \right)\right)\left(t\right)$ is not causal if its support is not contained in [0,+∞) (if g(t) is a continuous function, then it is enough to show that it is not equal to zero for a certain t≤0). Alternatively, g(t) is causal. The method seems to be very simple, but it may be really difficult to calculate the inverse Fourier transformation. In some cases, one knows the exact formula for the (inverse) Fourier transform for a given function or distribution. On the other hand, in numerous cases the exact formula is unknown.

In some cases, one can easily prove lack of causality by referring to the properties of the inverse Fourier transform. For instance, if the function G(ω) is an $L^{1}\left(R\right)$ function, then the inverse Fourier transform is a continuous function. Hence, it is enough to find a single point $t_{0}\in \left(-\infty ,0\right]$ such that $F^{-1}\left(G\left(\omega \right)\right)\left(t_{0}\right)\neq 0$, e.g., to show that $F^{-1}\left(G\left(\omega \right)\right)\left(0\right)\neq 0$. This idea may not be applied directly when G(ω) is an $L^{2}\left(R\right)$ function or a distribution which is not represented by an $L^{1}\left(R\right)$ function. With the definition of the Fourier transformation extended to the above-mentioned domains, one identifies the result up the sets of measure zero. In this case, without continuity of the result, showing that the inverse Fourier transform is non-zero at a single point does not prove lack of causality.

### 4.3. Holomorphic Extensions and K–K Relations

The classical perspective on causality is provided by the Titchmarsh theorem, which works for functions $g\in L^{2}\left(R\right)$ (see Theorem 1.6.1 in [4]):

**Theorem** **3.** *If a square-integrable function G(ω) fulfils one of the four conditions below, then it fulfils all four of them:*

*(i)*  *The inverse Fourier transform g(t) of G(ω) vanishes for t<0:*
g(t)=0(t<0).
*(ii)*  *G(v) is, for almost all v, the limit as $u\to 0^{+}$ of an analytic function $\tilde{G}\left(u+jv\right)$, which is holomorphic in the right half-plane and square integrable over any line parallel to the imaginary axis:*
$$\int_{-\infty }^{\infty }{\left|\tilde{G}\left(u+jv\right)\right|}^{2}dv<C\;\left(u>0\right).$$
*(iii)*  *ℜG and ℑG verify the first Plemelj formula:*
(83) $$ℜG\left(\omega \right)=-\frac{1}{\pi }{⨏}_{-\infty }^{+\infty }\frac{ℑG(\omega^{\prime })}{\omega^{\prime }-\omega }d\omega^{\prime }.$$
*(iv)*  *ℜG and ℑG verify the second Plemelj formula:*
(84) $$ℑG\left(\omega \right)=\frac{1}{\pi }{⨏}_{-\infty }^{+\infty }\frac{ℜG(\omega^{\prime })}{\omega^{\prime }-\omega }d\omega^{\prime }.$$

One should notice that the relations (83) and (84) hold in the sense of elements of the $L^{2}\left(R\right)$ space, i.e., the equalities hold for almost all ω∈R.

The relations (83) and (84) are also referred to as the K–K relations or the dispersion relations. This theorem delivers two aforementioned approaches to prove causality, i.e, searching for an appropriate holomorphic extension of G(ω) to the right-half plane, and proving the validity of the K–K relations (83) and (84).

If the function G(ω) is the Fourier transform of the real-valued function g(t), then it is *hermitian*, i.e., it has an even real part and an odd imaginary part. Therefore, the K–K relations (83) and (84) can be formulated for almost all ω∈R by the following integrals on (0,+∞):

(85) $$ℜG\left(\omega \right)=\frac{2}{\pi }{⨏}_{0}^{+\infty }\frac{\tau ℑG(\tau )}{\omega^{2}-\tau^{2}}d\tau$$

(86) $$ℑG\left(\omega \right)=-\frac{2\omega }{\pi }{⨏}_{0}^{+\infty }\frac{ℜG(\tau )}{\omega^{2}-\tau^{2}}d\tau .$$

It should be mentioned that the growth assumption in the case (ii) of Theorem 3 (i.e., that the maps $G_{\sigma }\left(\omega \right)=\tilde{G}\left(\sigma +j\omega \right)$ belong to $L^{2}\left(R\right)$ for all σ>0, and that all the norms $∥G_{\sigma }{∥}_{L^{2}\left(R\right)}$ are uniformly bounded by the same constant C>0) is vitally important. The sole existence of a holomorphic extension of the function G(ω) may not be sufficient for its causality. The case of $G\left(\omega \right)=e^{-\omega^{2}}$ is a good example. This function naturally extends to the holomorphic function $e^{s^{2}}$, where s=σ+jω, while the inverse Fourier transform $F^{-1}\left(G\right)=\frac{1}{\sqrt{2\pi }}e^{-t^{2}/4}$ is not a causal function. The uniform boundedness of the norm $∥G_{\sigma }{∥}_{L^{2}\left(R\right)}$ is the violated condition. It is because $|G_{\sigma }\left(\omega \right)|=e^{\sigma^{2}}e^{-\omega^{2}}$, thus, for σ→+∞, the norm can be arbitrarily large.

The general distributional version of the K–K relations, given as Theorem 5 in [25] (following Theorem 3.10 in [35]), is presented below. This version is a generalization of the well-known K–K relations with subtractions (as described in Section 1.7 of [4]). The procedure of subtractions works for such a Fourier transform G(ω), which is not necessarily in $L^{2}\left(R\right)$. However, when divided by some polynomial ${\left(\omega -p\right)}^{k}$ of degree *k*, it belongs to $L^{2}\left(R\right)$. The generalization towards distributions is sometimes required because the division itself can introduce a singularity, resulting in a function which is not locally square integrable (see the discussion in Section 4.2 in [25]). In the distributional version, it is not required that the division result, i.e., $F/{\left(j\omega \right)}^{k}$, is in $L^{2}\left(R\right)$, but it can be a distribution belonging to a class $D_{L^{q}}^{\prime }$ for some q∈(1,+∞), which can be broader than the functional space $L^{q}\left(R\right)$ (see Example 1 in [25]).

**Theorem** **4.** *Let us assume that $F={\left(j\omega \right)}^{k}G$, where $G\in D_{L^{q}}^{\prime }$. A necessary and sufficient condition that $F\in S^{\prime }$ should be the boundary value in the $S^{\prime }$ topology of a function $\tilde{F}\left(s\right)\in H^{+}$, i.e., that F and $\tilde{F}$ are, respectively, the Fourier and Laplace transforms of a distribution $f=F^{-1}\left(F\right)$, with supp(f)⊂[0,+∞), is that either*

(87) $$D^{k}F=\frac{1}{j\pi }F*D^{k}p.v.\frac{1}{\omega }$$

*or*

(88) $$F=\frac{{\left(j\omega \right)}^{k}}{j\pi }\left(G*p.v.\frac{1}{\omega }\right).$$

One should notice that (88) can be written with reference to the distributional version of the Hilbert transformation as

(89) $$F=\frac{{\left(j\omega \right)}^{k}}{j}H\left(G\right).$$

The other important detail hidden in this theorem is related to the growth condition on the holomorphic extension. One should remember that the condition $\tilde{F}\left(s\right)\in H^{+}$ means that $\tilde{F}$ is holomorphic in $C_{+}$, and that its growth is controlled by some polynomial as the condition (1) states.

A slightly less general version of Theorem 4, which can be used in the case of distribution *F* being represented as a locally integrable function, is formulated below.

**Theorem** **5** (Theorem 6, [25])**.** *Let us assume that k∈N∪{0} and F:R→C is a function such that $F\left(\omega \right)/{\left(j\omega \right)}^{k}$ is a locally integrable function of the growth at +∞ given by*

(90) $$\left|F\left(\omega \right)\right|\leq {C|\omega |}^{k-\varepsilon }$$

*for |ω|≥M, for some M>0, C>0 and for ε>0. Let us also assume that*

(91) $$F=\frac{{\left(j\omega \right)}^{k}}{j}H\left(\frac{F}{{\left(j\omega \right)}^{k}}\right)+P_{k-1}\left(\omega \right)$$

*where $P_{k-1}\left(\omega \right)$ is a certain polynomial of the degree k−1 at most. Then F is a causal transform.*

In some cases, the K–K relations can be evaluated for the transfer function logarithm. This attitude can result in sufficient conditions for causality.

**Theorem** **6** (Theorem 8, [25])**.** *Let us assume that the function L:R→C satisfies the following conditions:*

*(A1)*  *L:R→C is a locally integrable, hermitian function;*
*(A2)*  *$\left|L\left(\omega \right)\right|\leq {C|\omega |}^{\nu }$ for C>0, ν∈(0,1), |ω|≥M;*
*(A3)*  *the function K(ω)=L(ω)/(jω) is locally integrable;*
*(A4)*  *$K\left(\omega \right)=\frac{1}{j}H\left(K(\omega )\right)$;*
*(A5)*  *$V\left(\omega \right)=-\frac{ℜ(L(\omega ))}{\omega }=ℑ\left(K\left(\omega \right)\right)$ is a nonincreasing and nonnegative function for ω∈(0,+∞);*
*(A6)*  *$U\left(\omega \right)=\frac{1}{\omega }ℑ\left(L\left(\omega \right)\right)=ℜ\left(K\left(\omega \right)\right)$ is a nondecreasing function for ω∈(0,+∞).*

*Then $e^{L(\omega )}$ is a causal transform.*

The ‘holomorphic-extension’ approach can be generalized towards functions with a polynomial growth. The first theorem can be found in [35] as Theorem 2.7.

**Theorem** **7** (Theorem 2.7, [35])**.** *If $g\in S_{+}^{\prime }$ (i.e., g is a tempered distribution with a support in [0,+∞)), then its Laplace transform $L\left(g\right)\left(\sigma +j\omega \right)=F(g\left(t\right)e^{-\sigma t})\left(\omega \right)$ belongs to $H^{+}$ and L(g)(σ+jω)→F(g)(ω) in $S^{\prime }$ topology as $\sigma \to 0^{+}$. Conversely, if $\tilde{G}\left(s\right)=\tilde{G}\left(\sigma +j\omega \right)\in H^{+}$ and G(ω) is a limit of $\tilde{G}\left(\sigma +j\omega \right)$ as $\sigma \to 0^{+}$ in $S^{\prime }$ topology, then there exists $g\in S_{+}^{\prime }$ such that G=F(g) and $\tilde{G}=L\left(g\right)$.*

The theorem formulated above gives rise to practical sufficient conditions for causality of the transform G(ω).

**Theorem** **8.** *Let us assume that there exists a function $\tilde{G}\in H^{+}$, a locally integrable function G:R→R, a positive constant r>0 and a function f:R→R such that*

*(i)*  *f is locally integrable, with a growth in ±∞ limited by some polynomial, i.e., such that there exist M>0 and p∈N such that $\left|f\left(\omega \right)\right|\leq {\left|\omega \right|}^{p}$ for |ω|≥M (this implies that f represents a certain tempered distribution);*
*(ii)*  *the functions $G_{\sigma }:R\to R$ given by $G_{\sigma }\left(\omega \right)=\tilde{G}\left(\sigma +j\omega \right)$ are estimated by f, i.e., $|G_{\sigma }\left(\omega \right)|\leq |f\left(\omega \right)|$ for ω∈R and σ∈(0,r];*
*(iii)*  *$G_{\sigma }\left(\omega \right)\to G\left(\omega \right)$ as $\sigma \to 0^{+}$ for almost all ω∈R.*

*Then there exists $g\in S_{+}^{\prime }$ such that G=F(g) and $\tilde{G}=L\left(g\right)$.*

**Proof.**  This is a direct consequence of Theorem 7. Let us take any φ∈S and notice that

(92) $$\langle G_{\sigma },\varphi \rangle =\int_{-\infty }^{+\infty }G_{\sigma }\left(\omega \right)\varphi \left(\omega \right)d\omega .$$

The functions $G_{\sigma }$ are definitely continuous (as sections of holomorphic function), and hence locally integrable. Moreover, each of the integrals $\int_{-\infty }^{+\infty }\left|G_{\sigma }\left(\omega \right)\varphi \left(\omega \right)\right|d\omega$ and $\int_{-\infty }^{+\infty }\left|f\left(\omega \right)\right|$|φ(ω)|dω exists. Because

(93) $$|G_{\sigma }\left(\omega \right)\varphi \left(\omega \right)|\leq |f\left(\omega \right)||\varphi \left(\omega \right)|$$

for any σ∈(0,r] and almost all ω∈R, as well as the function $G_{\sigma }\left(\omega \right)\varphi \left(\omega \right)$ is (pointwise, almost everywhere) convergent to the function G(ω)φ(ω), we can refer to the Dominated Convergence Theorem (see, e.g., (2.206) in [5]) and state that

(94) $$\lim_{\sigma \to 0^{+}}\langle G_{\sigma },\varphi \rangle =\lim_{\sigma \to 0^{+}}\int_{-\infty }^{+\infty }G_{\sigma }\left(\omega \right)\varphi \left(\omega \right)d\omega =\int_{-\infty }^{+\infty }G\left(\omega \right)\varphi \left(\omega \right)d\omega =\langle G,\varphi \rangle .$$

It means that the tempered distributions $G_{\sigma }$ converge to *G* in $S^{\prime }$ topology. Thanks to Theorem 7, we know that G(ω) is a causal distribution. □

**Remark** **1.** *If the condition (ii) of the above theorem holds for r=+∞ (i.e., the estimate $|G_{\sigma }\left(\omega \right)|\leq |f\left(\omega \right)|$ holds for any ω∈R and σ∈(0,+∞)), then $\tilde{G}\in H^{+}$, i.e., the polynomial growth condition (1) holds.*

Furthermore, a more general version (with growth restrictions which are not necessarily polynomial) can be formulated as follows:

**Theorem** **9** (see [66] Theorem 3.8)**.** *Let us suppose that $G\in H(C_{+})$ satisfies the following:*

*(i)*  *for each $\rho_{0}>0$ function G restricted to the set $\left\{s\in C:ℜ\left(s\right)>\rho_{0}\right\}$ is of the order/type ≤(2,0);*
*(ii)*  *$b={lim sup}_{y\to +\infty }y^{-1}\left|\ln(G\left(y\right)\right|$ is finite;*
*(iii)*  *there exists R>0 such that, for all ρ∈(0,R], the function $G_{\rho }\left(\omega \right)=G\left(\rho +j\omega \right)$ satisfies $G_{\rho }\in S^{\prime }$.*

*Then there exists such a distribution $g\in D^{\prime }$ that supp(g)⊂[−b,+∞) and G is the Laplace transform of g.*

The set of distributional theorems presented in this section can be collected as a distributional version of the Titchmarsh theorem. It provides the conditions for the tempered distribution $f\in S^{\prime }$ to be supported in [0,+∞), due to the properties of its Fourier transform F=F(f). Let us mention that each of the tempered distributions F(ω) can be represented as $F\left(\omega \right)={\left(j\omega \right)}^{k}G\left(\omega \right)$, where $G\in D_{L^{q}}^{\prime }$ (see the discussion following Definition 3.2 in [35]).

**Theorem** **10.** *Let us assume that the tempered distribution $F\in S^{\prime }$ and $F={\left(j\omega \right)}^{k}G$, where $G\in D_{L^{q}}^{\prime }$. Then, if F fulfills one of the three conditions given below, it fulfills all of them:*

*(i)*  *for the distribution $f=F^{-1}\left(F\right)$, there is supp(f)⊂[0,+∞);*
*(ii)*  *the distribution F is the boundary value in the $S^{\prime }$ topology of a function $\tilde{F}\left(s\right)\in H^{+}$;*
*(iii)*  *the following relation*
(95) $$F=\frac{{\left(j\omega \right)}^{k}}{j}H\left(G\right)+P_{k-1}\left(\omega \right)$$
  *is satisfied for a certain polynomial $P_{k-1}\left(\omega \right)$ for the degree k−1 at most.*

**Proof.**  The equivalence (i)⇔(ii) is stated in Theorem 7 above (i.e., Theorem 2.7. in [35]). As one can see, (iii) is equivalent to (87), which is equivalent to (i) by Theorem 4. □

## 5. Causality Evaluations

### 5.1. Application of Paley-Wiener Theorem

**Example** **1.** *As it has been mentioned in Section 4.1 above, the Paley–Wiener theorem is a useful tool to detect the lack of causality. This is the case of frequency response of systems governed by the power law with an exponent ν>1. Let us take G(ω) as given by (73). One can see that $G\left(\omega \right)\in L^{2}\left(R\right)$ and*

(96) $$\left|\ln\left|G(\omega )\right|\right|=z\sqrt{\mu_{\gamma }\epsilon_{\beta }}\left|\cos\left(\frac{\pi \nu }{2}\right)\right|{\left|\omega \right|}^{\nu }.$$

*Then one can calculate the integral*

(97) $$\int_{-\infty }^{+\infty }\frac{{\left|\omega \right|}^{\nu }}{1+\omega^{2}}d\omega =+\infty$$

*for ν>1 and ν≠3,5,7,… (i.e., not being an odd positive integer). By the Paley–Wiener theorem, the transform G(ω) is definitely not causal. The case of ν=1 is obviously causal because $F^{-1}\left(e^{-jz\sqrt{\mu_{\gamma }\epsilon_{\beta }}\omega }\right)=\delta \left(t-z\sqrt{\mu_{\gamma }\epsilon_{\beta }}\right)$. The case of ν=3,5,7… is analyzed in Example 23 below.*

### 5.2. Calculations of Inverse Fourier Transformation

**Example** **2** (Dielectric model with constant permittivity (35))**.** *This model implies that electric susceptibility $\chi \left(\omega \right)=\epsilon_{r}-1$ is constant as well. This directly implies that*

(98) $$F^{-1}\left(\chi \left(\omega \right)\right)\left(t\right)=\left(\epsilon_{r}-1\right)\delta \left(t\right)$$

*is a causal distribution.*

**Example** **3** (Dielectric model with ohmic losses (38))**.** *This model is not valid for ω=0; hence it is extended in the distributional sense as follows:*

(99) $$\epsilon \left(\omega \right)=\epsilon_{0}\epsilon_{r}+\sigma \left(p.v.\frac{1}{j\omega }+\pi \delta \left(\omega \right)\right).$$

*This directly implies that*

(100) $$F^{-1}\left(\epsilon \left(\omega \right)\right)\left(t\right)=\epsilon_{0}\epsilon_{r}\delta \left(t\right)+\sigma u\left(t\right)$$

*is a causal distribution.*

**Example** **4** (Debye relaxation model (45))**.** *For this relaxation model, one can calculate the inverse Fourier transformation as follows [47] Section 9.2.1:*

(101) $$F^{-1}\left(\chi \left(\omega \right)\right)\left(t\right)=\frac{\Delta \epsilon_{r}}{\tau }e^{-\frac{t}{\tau }}u\left(t\right).$$

*For t < 0, $F^{-1}\left(\chi \left(\omega \right)\right)\left(t\right)=0$; hence (45) is a causal transform.*

**Example** **5** (Lorentz model (46))**.** *For this model, one can calculate the inverse Fourier transformation [47] Section 9.2.2. That is*

(102) $$\chi \left(\omega \right)=\frac{\Delta \epsilon_{r}\omega_{0}^{2}}{\omega_{0}^{2}+2j\omega \gamma -\omega^{2}}$$

*for $\omega_{0}>\gamma >0$. Then one obtains*

(103) $$F^{-1}\left(\chi \left(\omega \right)\right)\left(t\right)=\frac{\Delta \epsilon_{r}\omega_{0}^{2}}{\sqrt{\omega_{0}^{2}-\gamma^{2}}}e^{-\gamma t}\sin\left(\sqrt{\omega_{0}^{2}-\gamma^{2}}\;t\right)u\left(t\right).$$

*For t < 0, $F^{-1}\left(\chi \left(\omega \right)\right)\left(t\right)=0$; hence (46) is a causal transform.*

**Example** **6** (Lorentz in high-frequency limit-model (47))**.** *This model requires some explanation, which is important from the formal mathematical perspective. The function χ(ω), given by (47), is not locally integrable, so it may not be treated as a distribution. Hence we are not able to treat it as a Fourier transform of any function or distribution (in other way, it is not possible to find the inverse Fourier transformation thereof), which means that we are not able to formally ask whether it is a causal distribution or not. The natural way to treat it as a distribution is to use the * principal part* formalism (see, e.g., the discussion in Section 10.1 in [5]), which means that one can associate $\frac{1}{\omega^{2}}$ with the distribution $p.f.\frac{1}{\omega^{2}}$ given by*

$$\langle p.f.\frac{1}{\omega^{2}},\varphi \rangle =\lim_{\varepsilon \to 0^{+}}\left(\int_{-\infty }^{-\varepsilon }\frac{\varphi (x)}{x^{2}}dx+\int_{\varepsilon }^{+\infty }\frac{\varphi (x)}{x^{2}}dx-\frac{2\varphi (0)}{\varepsilon }\right).$$

*It might be shown that (in the distributional sense) $Dp.v.\frac{1}{\omega }=-p.f.\frac{1}{\omega^{2}}$. With this identification, i.e., treating $-\frac{\omega_{p}^{2}}{\omega^{2}}$ as $-\omega_{p}^{2}p.f.\frac{1}{\omega^{2}}$, one can discuss the causality of this model. That is, the inverse Fourier transform of*

(104) $$\chi \left(\omega \right)=-\omega_{p}^{2}p.f.\frac{1}{\omega^{2}}$$

*is given by*

(105) $$F^{-1}\left(\chi \left(\omega \right)\right)\left(t\right)=\frac{\omega_{p}^{2}}{2}t\mathrm{sgn}\left(t\right).$$

*For t < 0, $F^{-1}\left(\chi \left(\omega \right)\right)\left(t\right)\neq 0$; hence (104) is not a causal transform. However, this observation requires some comment as well (refer to the discussion about the Formulas (19a), (19b) and (20) in [67]). Let us notice that, for*

(106) $$f\left(t\right)=\omega_{p}^{2}tu\left(t\right),$$

*one obtains*

(107) $$F\left(f\right)\left(\omega \right)=-p.f.\frac{\omega_{p}^{2}}{\omega^{2}}+j\omega_{p}^{2}\pi \delta^{\prime }\left(\omega \right).$$

*Hence the model (47) can be causal if it is extended in the distributional sense as follows:*

(108) $$\chi \left(\omega \right)=-\omega_{p}^{2}p.f.\frac{1}{\omega^{2}}+j\omega_{p}^{2}\pi \delta^{\prime }\left(\omega \right).$$

*One is also referred to Example 20 given below, where causality of this model is discussed from the perspective of the K–K relations.*

**Example** **7** (Lorentz in high-frequency limit with static magnetic induction-model (48))**.** *One can notice that the Formula (48) is representable as*

(109) $$\chi \left(\omega \right)=\mp \frac{\omega_{p}^{2}}{\omega_{B}\omega }\pm \frac{\omega_{p}^{2}}{\omega_{B}}\frac{1}{\omega \pm \omega_{B}}$$

*where ω≠0 and $\omega \neq \mp \omega_{B}$. Because (109) includes singularities, we write it as*

(110) $$\chi \left(\omega \right)=\mp \frac{\omega_{p}^{2}}{\omega_{B}}p.v.\frac{1}{\omega }\pm \frac{\omega_{p}^{2}}{\omega_{B}}p.v.\frac{1}{\omega \pm \omega_{B}}.$$

*Then we use the following properties of the Fourier transformation:*

$$F\left(f\left(t\right)e^{j\omega_{B}t}\right)=F\left(\omega -\omega_{B}\right)$$

$$F\left(\frac{j}{2}\mathrm{sgn}\left(t\right)\right)=p.v.\frac{1}{\omega }.$$

*Hence one obtains*

$$F\left(\frac{j}{2}\mathrm{sgn}\left(t\right)e^{\mp j\omega_{B}t}\right)=\frac{1}{\omega \pm \omega_{B}}.$$

*Finally, the inverse Fourier transformation of (110) can easily be calculated as*

(111) $$F^{-1}\left(\chi \left(\omega \right)\right)\left(t\right)=\mp \frac{j\omega_{p}^{2}}{2\omega_{B}}\mathrm{sgn}\left(t\right)\pm \frac{j\omega_{p}^{2}}{2\omega_{B}}e^{\mp j\omega_{B}t}\mathrm{sgn}\left(t\right).$$

*However, (110) is not a causal transform. Furthermore, it is not a purely real function. Therefore, in order to obtain a causal transform, we extend (48) with distributional terms which are appropriate multiplicities of δ(ω) and $\delta (\omega \pm \omega_{B})$. That is*

(112) $$\chi \left(\omega \right)=\mp \frac{j\omega_{p}^{2}}{\omega_{B}}\left(p.v.\frac{1}{j\omega }+\pi \delta \left(\omega \right)\right)\pm \frac{j\omega_{p}^{2}}{\omega_{B}}\left(p.v.\frac{1}{j(\omega \pm \omega_{B})}+\pi \delta \left(\omega \pm \omega_{B}\right)\right).$$

*Then one obtains in the time domain*

(113) $$F^{-1}\left(\chi \left(\omega \right)\right)\left(t\right)=\mp \frac{j\omega_{p}^{2}}{\omega_{B}}u\left(t\right)\pm \frac{j\omega_{p}^{2}}{\omega_{B}}e^{\mp j\omega_{B}t}u\left(t\right).$$

*Although (113) is a causal function, its values are complex in the time domain. Hence it is not a physical formula for any susceptibility.*

*One is also referred to Example 21 given below, where causality of this model is discussed from the perspective of the K–K relations.*

**Example** **8** (Drude model (50))**.** *This formula is undefined for ω∈{0,jγ}. Hence we extend it in the distributional sense as follows:*

(114) $$\chi \left(\omega \right)=-p.v.\frac{\omega_{0}^{2}}{\omega^{2}-j\omega \gamma }+\frac{\pi \omega_{0}^{2}}{\gamma }\delta \left(\omega \right).$$

*For such a distributional extension, one can find the inverse Fourier transform (well known in literature [47] Section 9.2.3)*

(115) $$F^{-1}\left(\chi \left(\omega \right)\right)\left(t\right)=\frac{\omega_{0}^{2}}{\gamma }\left(1-e^{-\gamma t}\right)u\left(t\right).$$

*Hence, for t<0, $F^{-1}\left(\chi \left(\omega \right)\right)\left(t\right)=0$, which means that (114) is a causal transform.*

**Example** **9** (Djordjevic-Sarkar relationship for lossy dielectrics (41))**.** *This model is undefined for ω=0; hence we extend it in the distributional sense as follows:*

(116) $$\epsilon \left(\omega \right)=\epsilon_{0}\left(1+\frac{\Delta \epsilon_{r}^{\prime }}{log_{10}\frac{\omega_{2}}{\omega_{1}}}\frac{ln\frac{\omega_{2}+j\omega }{\omega_{1}+j\omega }}{ln10}+\frac{\sigma }{\epsilon_{0}}\left(p.v.\frac{1}{j\omega }+\pi \delta \left(\omega \right)\right)\right).$$

*Using the time-domain representation of the function (41) proposed in [6] (see Formula (6) therein with p=0), one can calculate*

(117) $$\epsilon \left(t\right)=\epsilon_{0}\delta \left(t\right)+\epsilon_{0}\left(\frac{\Delta \epsilon_{r}^{\prime }}{{log}_{10}\left(\frac{\omega_{2}}{\omega_{1}}\right)ln\left(10\right)}\frac{e^{-\omega_{2}t}-e^{-\omega_{1}t}}{t}+\frac{\sigma }{\epsilon_{0}}\right)u\left(t\right).$$

*It is a causal distribution.*

### 5.3. Applications of Holomorphic Extensions

**Example** **10** (Cole-Cole model (51))**.** *For α=2, the transform $\chi \left(\omega \right)=\frac{1}{1-\tau^{2}\omega^{2}}$ is not causal because $F^{-1}\left(\chi \right)=\frac{1}{2\tau }\mathrm{sgn}\left(t\right)\sin\left(t/\tau \right)$. If one wants to obtain a causal model for α=2, then it is necessary to add the distributional terms as follows:*

(118) $$\chi \left(\omega \right)=p.v.\frac{1}{1-\tau^{2}\omega^{2}}-\frac{j\pi }{2\tau }\left(\delta \left(\omega -\frac{1}{\tau }\right)-\delta \left(\omega +\frac{1}{\tau }\right)\right).$$

*Then one can calculate $F^{-1}\left(\chi \right)=\frac{1}{2\tau }u\left(t\right)\sin\left(t/\tau \right)$. Let us define the extension*

(119) $$X\left(s\right)=\frac{1}{1+{\left(s\tau \right)}^{\alpha }},\alpha \in \left(0,2\right).$$

*Let us denote $\theta =\mathrm{atan}\left(\frac{\omega }{\sigma }\right)\in \left(-\frac{\pi }{2},\frac{\pi }{2}\right)$, where s=σ+jω and σ>0. Because*

(120) $$s^{\alpha }={\left|s\right|}^{\alpha }e^{j\alpha \theta },$$

*one can notice that, for α∈(0,2), there is αθ∈(−π,π), so X(s) is holomorphic in $C_{+}$. Moreover, the functions $X_{\sigma }\left(\omega \right)=X\left(\sigma +j\omega \right)$ are given by*

(121) $$X_{\sigma }\left(\omega \right)=\frac{1}{1+\tau^{\alpha }{\left(\sigma +j\omega \right)}^{\alpha }}=$$

$$\frac{1+\tau^{\alpha }{\left(\sigma^{2}+\omega^{2}\right)}^{\alpha /2}\cos\left(\alpha \theta \right)-j\tau^{\alpha }{\left(\sigma^{2}+\omega^{2}\right)}^{\alpha /2}\sin\left(\alpha \theta \right)}{{\left(1+\tau^{\alpha }{\left(\sigma^{2}+\omega^{2}\right)}^{\alpha /2}\cos\left(\alpha \theta \right)\right)}^{2}+\tau^{2\alpha }{\left(\sigma^{2}+\omega^{2}\right)}^{\alpha }\sin^{2}\left(\alpha \theta \right)}.$$

*One can notice that*

(122) $$\lim_{\sigma \to 0^{+}}X_{\sigma }\left(\omega \right)=\chi \left(\omega \right)$$

*for all ω∈R. One can also notice that*

(123) $$|X_{\sigma }\left(\omega \right)|=\frac{1}{\sqrt{1+2\tau^{\alpha }{\left(\sigma^{2}+\omega^{2}\right)}^{\alpha /2}\cos\left(\alpha \theta \right)+\tau^{2\alpha }{\left(\sigma^{2}+\omega^{2}\right)}^{\alpha }}}.$$

*This expression can easily be estimated when α∈[0,1], implying that cos(αθ)≥0. Then one obtains*

(124) $$|X_{\sigma }\left(\omega \right)|\leq 1.$$

*The last estimate shows that all the functions $|X_{\sigma }\left(\omega \right)|$ are dominated by the function f(ω)=1. Hence, by Theorem 8, the function χ(ω) is a causal transform.*

*For α∈(1,2), one cannot be sure of the sign of cos(αθ). However, the derivations can be continued with a slightly different estimate. Let us review the denominator of (123)*

(125) $$\sqrt{1+2\tau^{\alpha }{\left|s\right|}^{\alpha }\cos\left(\alpha \theta \right)+\tau^{2\alpha }{\left|s\right|}^{2\alpha }}=\sqrt{{\left(1-|\tau s\right|}^{\alpha }{)}^{2}+2{\left|\tau s\right|}^{\alpha }\left(1+\cos\left(\alpha \theta \right)\right)}$$

*where $\left|s\right|=\sqrt{\sigma^{2}+\omega^{2}}$. For the fixed α∈(0,2), one obtains $\left|\alpha \theta \right|\leq \alpha \frac{\pi }{2}<\pi$. Hence the inequality is obtained*

(126) $$1+\cos\left(\alpha \theta \right)\geq c_{\alpha }>0$$

*for a certain positive constant $c_{\alpha }$. Two nonnegative terms are obtained in a denominator; hence one can write an estimate*

(127) $$|X_{\sigma }\left(\omega \right)|\leq \frac{1}{\sqrt{2c_{\alpha }}\tau {\left|s\right|}^{\alpha /2}}.$$

*Let us notice that α/2∈(0,1), so the function $f\left(\omega \right)=\frac{1}{\sqrt{2c_{\alpha }}\tau {\left|\omega \right|}^{\alpha /2}}$ is locally integrable and is bounded for |ω|→+∞. Hence it can be considered as a tempered distribution. Moreover, the inequality can be formulated as follows:*

(128) $$|X_{\sigma }\left(\omega \right)|\leq \frac{1}{\sqrt{2c_{\alpha }}\tau {\left|\sigma^{2}+\omega^{2}\right|}^{\alpha /4}}\leq f\left(\omega \right).$$

*One can also notice that $X\left(s\right)\in H^{+}$. It is definitely holomorphic in the right half-plane, so it is enough to show that it can be estimated by a polynomial in half planes $ℜs\geq \sigma_{0}>0$. Let s=σ+jω with $\sigma \geq \sigma_{0}$. Then one can estimate*

(129) $$\left|X\left(s\right)\right|\leq \frac{1}{\sqrt{2c_{\alpha }}\tau {\left|\sigma^{2}+\omega^{2}\right|}^{\alpha /4}}\leq \frac{1}{\sqrt{2c_{\alpha }}\tau \sigma_{0}^{\alpha /2}}$$

*by a constant. It proves that $X\left(s\right)\in H^{+}$; hence all the assumptions of Theorem 8 are satisfied. Therefore the Cole–Cole model (51) is causal for α∈(0,2).*

**Example** **11** (Cole-Davidson model (52))**.** *One can easily notice that, for β=1, the transform $\chi \left(\omega \right)=\frac{\Delta \epsilon_{r}}{1+j\omega \tau }=F\left(u\left(t\right)\frac{\Delta \epsilon_{r}}{\tau }e^{-t/\tau }\right)$ is a causal transform.*

*In the case of β∈(0,1), taking $X\left(s\right)=\frac{\Delta \epsilon_{r}}{{\left(1+\tau s\right)}^{\beta }}$, as well as denoting s=σ+jω and $\theta =\mathrm{atan}\left(\frac{\omega \tau }{1+\sigma \tau }\right)$, one obtains*

(130) $$X\left(s\right)=X\left(\sigma +j\omega \right)=\frac{\Delta \epsilon_{r}}{{\left({\left(1+\tau \sigma \right)}^{2}+\omega^{2}\tau^{2}\right)}^{\beta /2}}e^{-j\beta \theta }.$$

*Therefore one can state that $|X\left(s\right)|\leq |\Delta \epsilon_{r}|$ for any $s\in C_{+}$. It means that both $X\in H^{+}$ and $|X_{\sigma }\left(\omega \right)|\leq f\left(\omega \right)$ for a locally integrable (actually constant) function $f\left(\omega \right)=|\Delta \epsilon_{r}|$. Hence, all the assumptions of Theorem 8 are satisfied and the transform X(ω) is causal.*

**Example** **12** (Havriliak-Negami model (53))**.** *This model can naturally be extended towards the function $X\left(s\right)=\frac{\Delta \epsilon_{r}}{{\left(1+{\left(s\tau \right)}^{\alpha }\right)}^{\beta }}$, holomorphic in the right half-plane $C_{+}$. Let us denote $\theta =\mathrm{atan}\left(\frac{\omega }{\sigma }\right)\in \left(-\frac{\pi }{2},\frac{\pi }{2}\right)$, similarly as for the Cole–Cole model. Let us also denote s=σ+jω. It gives*

(131) $$\left|X\left(s\right)\right|=\frac{|\Delta \epsilon_{r}|}{{\left(1+2\tau^{\alpha }{\left|s\right|}^{\alpha }\cos\left(\theta \alpha \right)+\tau^{2\alpha }{\left|s\right|}^{2\alpha }\right)}^{\beta /2}}\leq \left|\Delta \epsilon_{r}\right|,$$

*because cos(θα)≥0. Hence one obtains the same estimate as in the previously considered models. Similarly as before, by Theorem 8, one can state that the transform is causal.*

**Example** **13** (Raicu model (54))**.** *This model has natural holomorphic extension to $C_{+}$, given by*

(132) $$X\left(s\right)=\frac{\Delta }{{\left({\left(s\tau \right)}^{\gamma }+{\left(s\tau \right)}^{\alpha }\right)}^{\beta }}.$$

*It leads to*

(133) $$\left|X\left(s\right)\right|=\frac{\Delta }{{\left({\left|\tau s\right|}^{2\gamma }+{2|\tau s|}^{\gamma +\alpha }\cos\left(\theta \left(\gamma -\alpha \right)\right)+{\left|\tau s\right|}^{2\alpha }\right)}^{\beta /2}}$$

*where s=σ+jω and $\theta =\mathrm{atan}\left(\frac{\omega }{\sigma }\right)\in \left[-\frac{\pi }{2},\frac{\pi }{2}\right]$. Because $\theta \left(\gamma -\alpha \right)\in \left(-\frac{\pi }{2},\frac{\pi }{2}\right)$ and cos(θ(γ−α))≥0, the following estimate is obtained*

(134) $$\left|X\left(s\right)\right|\leq \frac{\Delta }{{\left|s\right|}^{\gamma \beta }}$$

*(one can take the exponent αβ as well). In this model, one cannot use the trivial estimate by the constant function equal to |Δ|, so let us first prove that $X\left(s\right)\in H^{+}$, i.e., it can be estimated by a polynomial in any half plane $ℜs\geq \sigma_{0}>0$. If one assumes $ℜs\geq \sigma_{0}$, then also $\left|s\right|\geq \sigma_{0}$, implying that*

(135) $$\left|X\left(s\right)\right|\leq \frac{\Delta }{{\left|s\right|}^{\gamma \beta }}\leq \frac{\Delta }{\sigma_{0}^{\gamma \beta }}.$$

*Hence it is estimated by a constant in any half-plane $ℜs\geq \sigma_{0}>0$.*

*Let us first observe that, if α=β=γ=1, the Raicu model gives $\chi \left(\omega \right)=\frac{\Delta }{j2\omega \tau }$. In this case, $F^{-1}\left(\chi \left(\omega \right)\right)=\frac{\Delta }{4\tau }\mathrm{sgn}\left(t\right)$ is not a causal function. However, if it is extended by the distributional term, i.e., $\chi \left(\omega \right)=\frac{\Delta }{j2\omega \tau }+\frac{\pi \Delta }{2\tau }\delta \left(\omega \right)$, then one obtains a causal transform $F^{-1}\left(\chi \left(\omega \right)\right)=\frac{\Delta }{4\tau }u\left(t\right)$.*

*Let us now assume that at least one of the constants α,β,γ belongs to [0,1). Then let us assume that γβ∈[0,1) in the estimate (134). Let us take the locally integrable function $f\left(\omega \right)=\frac{1}{{\left|\omega \right|}^{\gamma \beta }}$. This function can obviously be considered as a tempered distribution (it is bounded by a constant as |ω|→+∞). Moreover, for any σ>0, one can notice that*

(136) $$|X_{\sigma }\left(\omega \right)|=|X\left(\sigma +j\omega \right)|\leq \frac{\Delta }{|\sigma^{2}+\omega^{2}{|}^{\gamma \beta /2}}\leq f\left(\omega \right).$$

*Hence, all the assumptions of Theorem 8 are satisfied and the transform χ(ω) given by (54) is causal for all α,β,γ∈[0,1], excluding the special case of α=β=γ=1.*

**Example** **14** (Lorentz model in FO generalisation (49))**.** *This model has a natural holomorphic extension given by*

(137) $$X\left(s\right)=\frac{{\left(\frac{\omega_{p}}{\omega_{0}}\right)}^{2}}{1+2\gamma {\left(\frac{s}{\omega_{0}}\right)}^{\alpha }+{\left(\frac{s}{\omega_{0}}\right)}^{2\alpha }},\alpha \in \left(0,1\right].$$

*The open question is whether this extension is well-defined over $C_{+}$. First, let us observe that the quadratic equation*

(138) $$1+2\gamma z+z^{2}=0$$

*with an unknown $z={\left(\frac{s}{\omega_{0}}\right)}^{\alpha }$ has the following solutions*

(139) $$z=-\gamma \pm \sqrt{\gamma^{2}-1}.$$

*Hence, no matter if $\gamma^{2}-1$ is positive or not, the real part of the solution z is negative. It means that, for all $z\in C_{+}$, the denominator of the function given by (137) is non-zero. If $s\in C_{+}$, then also $z={\left(\frac{s}{\omega_{0}}\right)}^{\alpha }\in C_{+}$.*

*Hence the extension (137) is well-defined over the entire half-plane $C_{+}$. Now we are going to check the assumptions of Theorem 8. First, since the modulus of the denominator $\left|1+2\gamma {\left(\frac{s}{\omega_{0}}\right)}^{\alpha }+{\left(\frac{s}{\omega_{0}}\right)}^{2\alpha }\right|$ is bounded from below by some constant C>0 in the closed right half-plane $\bar{C_{+}}$, one can write the estimate*

(140) $$|X_{\sigma }\left(\omega \right)|=|X\left(\sigma +j\omega \right)|\leq \frac{1}{C}{\left(\frac{\omega_{p}}{\omega_{0}}\right)}^{2}=f\left(\omega \right)$$

*for any σ≥0 and ω∈R. Because the pointwise convergence $X_{\sigma }\left(\omega \right)=\chi \left(\omega \right)$ as $\sigma \to 0^{+}$ is obvious, by Theorem 8, the function χ(ω) is a causal transform.*

Now, let us take a look at the transfer functions which are derived for the formulation of time-fractional electrodynamics based on the RS vector.

**Example** **15** (Plane-wave and spherical symmetries of solutions to diffusion-wave equation formulated based on RS vector-models (78) and (79))**.** *For the functions $G_{z}$ and $G_{R}$, given by (78) and (79), respectively, the appropriate holomorphic extensions exist for β∈(0,1), i.e.,*

(141) $$G_{z}\left(\sigma +j\omega \right)=G_{z}\left(s\right)=e^{-z\sqrt{\mu_{\beta }\epsilon_{\beta }}s^{\beta }}$$

(142) $$G_{R}\left(\sigma +j\omega \right)=G_{R}\left(s\right)=\frac{1}{R}e^{-R\sqrt{\mu_{\beta }\epsilon_{\beta }}s^{\beta }}.$$

*The assumptions of the Titchmarsh Theorem 3 are satisfied in the point (ii) for both functions; hence it directly proves their causality.*

**Example** **16** (Cylindrical symmetry of solution to diffusion-wave equation formulated based on RS vector-model (80))**.** *The function $G_{r}$, given by (80), does not belong to $L^{2}\left(R\right)$. It stems from the asymptotics of the Bessel function $J_{0}$ given by*

(143) $$J_{0}\left(z\right)=\sqrt{\frac{2}{\pi z}}\cos\left(z-\frac{\pi }{4}\right)+e^{\left|ℑz\right|}O\left({\left|z\right|}^{-3/2}\right)$$

*valid for z∈C such that argz∈(−π,π) as |z|→+∞ (see Formula 9.2.1 in [68]; more details are given in Chapter VII in [69]). A natural holomorphic extension to the right half-plane for $G_{r}\left(\omega \right)$ is given by*

(144) $$G_{r}\left(\sigma +j\omega \right)=G_{r}\left(s\right)=J_{0}\left(js^{\beta }r\sqrt{\mu_{\beta }\epsilon_{\beta }}\right).$$

*The behavior in infinity described by (143) does not allow one to state that the function $J_{0}\left(js^{\beta }r\sqrt{\mu_{\beta }\epsilon_{\beta }}\right)$ has a polynomial growth for ℜs≤ρ, and a positive ρ>0. That is why, when trying to prove the causality of the operator, one should refer to the stronger Theorem 9 and treat the function $G_{r}\left(\omega \right)$ as a tempered distribution. The condition (i) of above-mentioned Theorem 9 is equivalent to the statement that, for any ε>0, such $C=C(\varepsilon ,\rho_{0})>0$ exists that*

(145) $$\ln|G\left(s\right)|\leq \ln C+{\varepsilon |s|}^{2}.$$

*As one can see, due to the estimate (143), the modulus $|G_{r}\left(s\right)|$ can be estimated by $Ce^{{\left|s\right|}^{\beta }}$ as |s|→+∞; hence the estimate (145) is satisfied. Moreover, taking y∈R, one can write*

(146) $$G_{r}\left(y\right)=J_{0}\left(jy^{\beta }\right)$$

*and because of the trivial estimate*

(147) $$|\ln\left(J_{0}\left(jy^{\beta }\right)\right)|\leq \left|\ln(|J_{0}\left(jy^{\beta }\right)|)\right|+\pi$$

*one can focus on the estimate of $|J_{0}\left(jy^{\beta }\right)|$. Looking at the terms on the right-hand side of (143), one can see that the dominating term is of the order $e^{{A|s|}^{\beta }}$ for some constant A>0. Hence one obtains*

(148) $$\underset{y\to +\infty }{lim sup}y^{-1}|\ln(G_{r}\left(y)\right)|=0.$$

*On the other hand, by the Hankel’s Asymptotic Expansions (see [68] Equation (9.2.5) and [69] Section 7.3), the function $J_{0}\left(js^{\beta }r\sqrt{\mu_{\beta }\epsilon_{\beta }}\right)$ is bounded as |s|→+∞. Hence, the function (144) satisfies all the assumptions of Theorem 9, so it is also a causal transform.*

### 5.4. Applications of K–K Relations

**Example** **17** (Westerlund relationship for FO capacitors [42,43] and power-law relationship for porous media [45]-models (42) and (44))**.** *Both Equations (42) and (44) are in the form*

(149) $$\chi \left(\omega \right)=\frac{C}{{\left(j\omega \right)}^{\alpha }}-1$$

*for certain constants C>0 and α∈(0,1). We are going to show that the function*

(150) $$F\left(\omega \right)=\frac{1}{{\left(j\omega \right)}^{\alpha }}$$

*is a causal transform (subtracting 1 does not influence causality, since it is a transform of the Dirac delta, i.e., a causal distribution). One can see that*

$${\left(j\omega \right)}^{-\alpha }={\left|\omega \right|}^{-\alpha }\cos\left(\frac{\pi }{2}\alpha \right)-j{\left|\omega \right|}^{-\alpha }\sin\left(\frac{\pi }{2}\alpha \mathrm{sgn}\left(\omega \right)\right).$$

*Let us refer to Theorem 5 for k=0. As F(ω) is locally integrable and satisfies growth conditions in ±∞, it is sufficient to check the K–K relation (91), i.e.,*

(151) $$F=\frac{1}{j}H\left(F\right).$$

*Let us calculate the Hilbert transforms*

(152) $$H\left({\left|\omega \right|}^{-\alpha }\cos\left(\frac{\pi }{2}\alpha \right)\right)=\frac{\cos(\frac{\pi }{2}\alpha )}{\pi }{⨏}_{-\infty }^{+\infty }\frac{{\left|\tau \right|}^{-\alpha }}{\omega -\tau }d\tau$$

*and*

(153) $$H\left({\left|\omega \right|}^{-\alpha }\sin\left(\frac{\pi }{2}\alpha \mathrm{sgn}\left(\omega \right)\right)\right)=\frac{\sin(\frac{\pi }{2}\alpha )}{\pi }{⨏}_{-\infty }^{+\infty }\frac{{\mathrm{sgn}\left(\tau \right)|\tau |}^{-\alpha }}{\omega -\tau }d\tau .$$

*Let us refer to a certain integral formula taken from (3.241.3) in [70] (valid for p<q)*

(154) $${⨏}_{0}^{\infty }\frac{x^{p-1}}{1-x^{q}}dx=\frac{\pi }{q}\cot\frac{p\pi }{q}.$$

*Taking q=2 and p=−α+1<2, as well as applying the substitution x=τ/ω, one obtains*

(155) $${⨏}_{0}^{\infty }\frac{{\left|\tau \right|}^{-\alpha }}{\omega^{2}-\tau^{2}}d\tau =\omega^{-\alpha -1}{⨏}_{0}^{\infty }\frac{x^{-\alpha }}{1-x^{2}}dx=\omega^{-\alpha -1}\frac{\pi }{2}\cot\frac{(1-\alpha )\pi }{2}.$$

*Similarly, taking p=−α+2<2, one obtains*

(156) $${⨏}_{0}^{\infty }\frac{{\left|\tau \right|}^{-\alpha +1}}{\omega^{2}-\tau^{2}}d\tau =\omega^{-\alpha }{⨏}_{0}^{\infty }\frac{x^{-\alpha +1}}{1-x^{2}}dx=\omega^{-\alpha }\frac{\pi }{2}\cot\frac{(2-\alpha )\pi }{2}.$$

*Now, we take the Formula (152) and convert it to the integral over (0,+∞)*

(157) $${⨏}_{-\infty }^{+\infty }\frac{{\left|\tau \right|}^{-\alpha }}{\omega -\tau }d\tau =2\omega {⨏}_{0}^{+\infty }\frac{\tau^{-\alpha }}{\omega^{2}-\tau^{2}}d\tau =$$

$$2\omega^{-\alpha }\frac{\pi }{2}\cot\frac{(1-\alpha )\pi }{2}.$$

*It means that*

(158) $$H\left({\left|\omega \right|}^{-\alpha }\cos\left(\frac{\pi }{2}\alpha \right)\right)=\frac{\cos(\frac{\pi }{2}\alpha )}{\pi }2\omega^{-\alpha }\frac{\pi }{2}\cot\frac{(1-\alpha )\pi }{2}=\sin\left(\frac{\pi }{2}\alpha \right)\omega^{-\alpha }$$

*and the real parts on both sides of (151) coincide. Now, we take the Formula (153) and convert it to the integral over (0,+∞)*

(159) $${⨏}_{-\infty }^{+\infty }\frac{{\mathrm{sgn}\left(\tau \right)|\tau |}^{-\alpha }}{\omega -\tau }d\tau =2{⨏}_{0}^{+\infty }\frac{\tau^{-\alpha +1}}{\omega^{2}-\tau^{2}}d\tau =$$

$$2\omega^{-\alpha }\frac{\pi }{2}\cot\frac{(2-\alpha )\pi }{2}.$$

*It means that*

(160) $$H\left({\left|\omega \right|}^{-\alpha }\sin\left(\frac{\pi }{2}\alpha \mathrm{sgn}\left(\omega \right)\right)\right)=\frac{\sin(\frac{\pi }{2}\alpha )}{\pi }2\omega^{-\alpha }\frac{\pi }{2}\cot\frac{(2-\alpha )\pi }{2}=-\cos\left(\frac{\pi }{2}\alpha \right)\omega^{-\alpha },$$

*which proves that the imaginary parts of (151) coincide as well. It completes the proof that all the assumptions of Theorem 5 are satisfied; hence the transform (149) is causal. One should notice that the solutions obtainable for various power-law media and time-fractional electrodynamics may not be relativistically causal. That is, for the time-fractional diffusion-wave equation, the propagation velocity of a disturbance is infinite, but its fundamental solution possesses a maximum which disperses with a finite velocity [63].*

**Example** **18.** *Let $G\left(\omega \right)=\frac{1}{j\omega }$. This is the function which is not locally integrable, so not in $L^{2}\left(R\right)$, but can be identified with a distribution $p.v.\frac{1}{j\omega }$, which is in $D_{L^{2}}$. Then the K–K relations (87) can be interpreted for k=0 as*

(161) $$G=\frac{1}{j\pi }G*p.v.\frac{1}{\omega }=\frac{1}{j}H\left(G\right).$$

*Let us observe that, by (20), there is*

$$\frac{1}{j}H\left(G\right)=-\frac{\pi }{j^{2}}\delta \left(\omega \right)=\pi \delta \left(\omega \right).$$

*Hence the relation (161) is not satisfied. On the other hand, when the modified transform is taken*

$$G_{c}\left(\omega \right)=p.v.\frac{1}{j\omega }+\pi \delta \left(\omega \right)$$

*one can see that (referring to (19))*

$$\frac{1}{j}H\left(G_{c}\right)=\pi \delta \left(\omega \right)+\frac{\pi }{j}p.v.\frac{\pi }{\omega }=G_{c}\left(\omega \right).$$

*However, it is well-known that $F^{-1}\left(G\left(\omega \right)\right)=\frac{1}{2}\mathrm{sgn}\left(t\right)$, which is not a causal function, and $F^{-1}\left(G_{c}\left(\omega \right)\right)=\frac{1}{2}\mathrm{sgn}\left(t\right)+\frac{1}{2}=u\left(t\right)$, which is a causal function. This fact can seem to be slightly paradoxical. It is because the transform $p.v.\frac{1}{j\omega }$ is closely related to the operator*

(162) $$f\mapsto \int_{-\infty }^{t}f\left(s\right)ds$$

*assigning an integrable function its primitive. It is natural to consider this operator as a causal operator, as it converts causal integrable functions to causal locally integrable functions. The problem is that multiplication by the function $\frac{1}{j\omega }$ does not always correspond to integration in the time domain. Unfortunately, the time-domain integration operator, applied to functions from the space $L^{1}\left(R\right)$ or $L^{2}\left(R\right)$ or to functions from the Schwartz space S, does not always give a function from one of these spaces. The condition necessary to obtain the antiderivative operator $\int_{-\infty }^{t}f\left(s\right)ds$ acting to any of these spaces is that $\int_{-\infty }^{+\infty }f\left(s\right)ds=0$, and it is not always satisfied. Under this assumption, one can write*

(163) $$F\left(\int_{-\infty }^{t}f\left(s\right)ds\right)=\frac{1}{j\omega }F\left(\omega \right)$$

*where F(ω)=F(f)(ω). This is the only case when one can associate the multiplication by the function $\frac{1}{j\omega }$ with an integration operator. In the more general case of $f\in L^{1}\left(R\right)$ and $\int_{-\infty }^{+\infty }f\left(s\right)ds=F\left(0\right)\in R$ (not necessarily equal to zero), one obtains*

(164) $$F\left(\int_{-\infty }^{t}f\left(s\right)ds\right)=\frac{1}{j\omega }F\left(\omega \right)+\pi F\left(0\right)\delta \left(\omega \right).$$

*However, this formula does not work for all the tempered distributions. For instance, let us take f(t)=u(t), i.e., the Heaviside step function. Then*

$$g\left(t\right)=\int_{-\infty }^{t}f\left(s\right)ds=tu\left(t\right)$$

*and $F\left(g\right)\left(\omega \right)=D\left(p.v.\frac{1}{j\omega }\right)+i\pi \delta^{\prime }\left(\omega \right)$, where $\delta^{\prime }$ denotes the distributional derivative of the Dirac delta, and D(·) denotes the distributional derivative. On the other hand, $F\left(\omega \right)=F\left(f\right)\left(\omega \right)=p.v.\frac{1}{j\omega }+\pi \delta \left(\omega \right)$ and none of the Formulas (163), nor (164) can be correct (actually, these are not even well defined, due to the fact that the multiplication of distributions is not well-defined in general).*

**Example** **19.** *Let us fix a∈R, and check when $G\left(\omega \right)=e^{-ja\omega }$ is a causal transform. The answer is obvious, because G(ω)=F(δ(t−a)), which is a causal distribution iff a∈[0,+∞). However, we are going to look at the Formula (87)*

(165) $$D^{k}G=\frac{1}{j\pi }G*D^{k}p.v.\frac{1}{\omega }$$

*for k=1 (the value of k is selected as the minimal natural number, such that $\frac{e^{-ja\omega }}{\omega^{k}}\in D_{L^{2}}^{\prime }$).*

*The left-hand side of the above inequality is obviously $DG\left(\omega \right)=-jae^{-ja\omega }$. On the other hand, one can write the right-hand side as*

(166) $$\frac{1}{j\pi }G*Dp.v.\frac{1}{\omega }=\frac{1}{j\pi }D\left(G*p.v.\frac{1}{\omega }\right).$$

*Let us now calculate the convolution for the test function $\varphi \in D_{L^{2}}$*

(167) $$\langle e^{-ja\omega }*p.v.\frac{1}{\omega },\varphi \rangle =\int_{-\infty }^{+\infty }e^{-ja\omega }\lim_{\varepsilon \to 0^{+}}\left(\int_{|\xi |>\varepsilon }\frac{\varphi (\xi +\omega )}{\xi }d\xi \right)d\omega =$$

$$\int_{-\infty }^{+\infty }e^{-ja\omega }\lim_{\varepsilon \to 0^{+}}\left(\int_{\varepsilon }^{+\infty }\frac{\varphi (\omega +\xi )-\varphi (\omega -\xi )}{\xi }d\xi \right)d\omega =$$

$$\lim_{\varepsilon \to 0^{+}}\int_{\varepsilon }^{+\infty }\frac{1}{\xi }\int_{-\infty }^{+\infty }\left(\varphi (\omega +\xi )-\varphi (\omega -\xi )\right)e^{-ja\omega }d\omega d\xi =$$

$$\lim_{\varepsilon \to 0^{+}}\int_{\varepsilon }^{+\infty }\frac{1}{\xi }\left(\int_{-\infty }^{+\infty }\varphi \left(t\right)e^{-jat}dt\right)\left(e^{ja\xi }-e^{-ja\xi }\right)d\xi =$$

$$2j\left(\int_{-\infty }^{+\infty }\varphi \left(t\right)e^{-jat}dt\right)\int_{0}^{+\infty }\frac{\sin(a\xi )}{\xi }d\xi =\;j\pi \mathrm{sgn}\left(a\right)\int_{-\infty }^{+\infty }\varphi \left(t\right)e^{-jat}dt=\langle j\pi \mathrm{sgn}\left(a\right)e^{-jat},\varphi \rangle .$$

*It means that $\frac{1}{j\pi }G*p.v.\frac{1}{\omega }=\mathrm{sgn}\left(a\right)e^{-ja\omega }$ and*

(168) $$\frac{1}{j\pi }D\left(G*p.v.\frac{1}{\omega }\right)=-ja\mathrm{sgn}\left(a\right)e^{-ja\omega }.$$

*Hence, both sides of (166) coincide iff asgn(a)=a, i.e., for a∈[0,+∞).*

Having reviewed two abstract cases, let us look at some of the models from the perspective of the K–K relations.

**Example** **20** (Lorentz in high-frequency limit-model (47))**.** *Because we treat the function $\frac{1}{\omega^{2}}$ as a distribution $p.f.\frac{1}{\omega^{2}}$ (see the discussion in Example 6), one can write*

(169) $$\chi \left(\omega \right)=\omega_{p}^{2}D\left(p.v.\frac{1}{\omega }\right).$$

*It means that $\chi \left(\omega \right)\in D_{L^{q}}^{\prime }$ as a derivative of the distribution from $D_{L^{q}}^{\prime }$. Then it means that the K–K relations (87) should be taken for k=0. Let us consider*

$$\frac{1}{j}H\left(\chi \left(\omega \right)\right)=\frac{\omega_{p}^{2}}{j}HD\left(p.v.\frac{1}{\omega }\right)=\frac{\omega_{p}^{2}}{j}DH\left(p.v.\frac{1}{\omega }\right)=$$

$$j\pi \omega_{p}^{2}\delta^{\prime }\left(\omega \right)\neq \chi \left(\omega \right).$$

*In these derivations, the Formulas (18) and (20) are used. Hence one can conclude that the transform χ(ω) is not causal. However, as previously, one can notice that, by adding a singular term to χ(ω), a causal transform is obtained. That is*

(170) $$\chi \left(\omega \right)=-p.f.\frac{\omega_{p}^{2}}{\omega^{2}}+j\pi \omega_{p}^{2}\delta^{\prime }\left(\omega \right)$$

*is a causal transform.*

**Example** **21** (Lorentz in high-frequency limit with static magnetic induction–model (48))**.** *As before, one can see that the Formula (48) is representable as*

(171) $$\chi \left(\omega \right)=\mp \frac{\omega_{p}^{2}}{\omega_{B}\omega }\pm \frac{\omega_{p}^{2}}{\omega_{B}}\frac{1}{\omega \pm \omega_{B}}.$$

*Let us check the K–K relations for this function. Since $\chi \left(\omega \right)\in D_{L^{2}}^{\prime }$, one can refer to (87) for k=0; hence one can directly refer to (161). By the Formulas (20) and (21), one can notice that*

(172) $$\frac{1}{j}H\left(\chi \left(\omega \right)\right)=\frac{1}{j}H\left(\mp \frac{\omega_{p}^{2}}{\omega_{B}\omega }\pm \frac{\omega_{p}^{2}}{\omega_{B}}\frac{1}{\omega \pm \omega_{B}}\right)=\;j\pi \left(\mp \frac{\omega_{p}^{2}}{\omega_{B}}\delta \left(\omega \right)\pm \frac{\omega_{p}^{2}}{\omega_{B}}\delta \left(\omega \pm \omega_{B}\right)\right)\neq \chi \left(\omega \right).$$

*It implies that χ(ω) is not a causal transform. However, it can be concluded that adding singular terms in order to obtain*

(173) $$\chi \left(\omega \right)=\mp \frac{\omega_{p}^{2}}{\omega_{B}\omega }\pm \frac{\omega_{p}^{2}}{\omega_{B}}\frac{1}{\omega \pm \omega_{B}}+j\pi \left(\mp \frac{\omega_{p}^{2}}{\omega_{B}}\delta \left(\omega \right)\pm \frac{\omega_{p}^{2}}{\omega_{B}}\delta \left(\omega \pm \omega_{B}\right)\right)$$

*results in a causal transform.*

### 5.5. How to Prove Lack of Causality?

Obviously, one can prove that an appropriate holomorphic extension $\tilde{G}\left(s\right)$ of the transform G(ω) *does not* exist, but it does not look like an easy task. One should return to the K–K relations instead, and prove that one of the equalities (83) or (84) (or in the case of hermitian transforms (85) or (86)) is not satisfied in $L^{2}\left(R\right)$. Because the equalities are given between the elements of $L^{2}\left(R\right)$ space, it means that violation of any of the conditions (83) and (84) for the transform G(ω) in a single point does not prove that the transform is not causal in general. Equations (83) and (84) are in $L^{2}$ sense; hence such equalities are valid almost everywhere. Fortunately, in certain cases, it occurs that the relations (83) and (84) are valid for all ω∈R.

We now provide the theorem stated by Wood in 1929 ([71] Theorem I, see also [5] Section 3.4.1):

**Theorem** **11** (Wood, [71] Theorem I)**.** *Assuming that f,g:R→R are such functions that*

*(i)*  *$g\left(x\right)=\frac{1}{\pi }{⨏}_{-\infty }^{+\infty }\frac{f(t)}{x-t}dt$;*
*(ii)*  *the integrals $\int_{M}^{+\infty }\frac{f(t)}{t}dt$ and $\int_{-\infty }^{-M}\frac{f(t)}{t}dt$ exist for certain M>0;*
*(iii)*  *f is locally Hölder continuous of the order α∈(0,1).*

*Then the function g is also locally Hölder continuous with an exponent α and*

(174) $$f\left(x\right)=-\frac{1}{\pi }{⨏}_{-\infty }^{+\infty }\frac{g(t)}{x-t}dt$$

*holds for all x∈R.*

Hence, for the Hölder continuous functions ℜG(ω) or ℑG(ω), with a behavior in ±∞ as required by Theorem 11, violation of any of the relations (83) or (84) in a single point proves that they do not hold in $L^{2}\left(R\right)$. This attitude is used in the following example:

**Example** **22.** *In [26], the transfer function induced by the two-sided fractional derivative introduced by Ortigueira and Machado (see [72,73]) is considered. It results in the following transfer function:*

(175) $$G_{\Theta ,\nu }\left(\omega \right)=e^{-\mathrm{sgn}\left(\cos\left(\frac{\pi }{2}\nu \Theta \right)\right)\frac{z}{c_{\mu \epsilon }}{\left(j\omega \right)}^{\nu }e^{j\frac{\pi }{2}\nu \left(\Theta -1\right)\mathrm{sgn}\left(\omega \right)}}=e^{-\mathrm{sgn}\left(\cos\left(\frac{\pi }{2}\nu \Theta \right)\right)\frac{z}{c_{\mu \epsilon }}{\left|\omega \right|}^{\nu }e^{j\frac{\pi }{2}\nu \Theta \mathrm{sgn}\left(\omega \right)}}.$$

*Hence one obtains*

(176) $$ℜG_{\Theta ,\nu }\left(\omega \right)=e^{-\frac{z}{c_{\mu \epsilon }}|\cos\left(\frac{\pi }{2}\nu \Theta \right){\left||\omega \right|}^{\nu }}\cos\left(\frac{z}{c_{\mu \epsilon }}{\left|\omega \right|}^{\nu }\sin\left(\frac{\pi }{2}\nu \Theta \right)\right)$$

(177) $$ℑG_{\Theta ,\nu }\left(\omega \right)=\;e^{-\frac{z}{c_{\mu \epsilon }}|\cos\left(\frac{\pi }{2}\nu \Theta \right){\left||\omega \right|}^{\nu }}\sin\left(-\mathrm{sgn}\left(\cos\left(\frac{\pi }{2}\nu \Theta \right)\right)\frac{z}{c_{\mu \epsilon }}{\left|\omega \right|}^{\nu }\sin\left(\frac{\pi }{2}\nu \Theta \mathrm{sgn}\left(\omega \right)\right)\right).$$

*As it is shown in Lemma 2 in [26], for this transfer function, as well as for ν∈(0,1) and $\frac{1}{2}\left(\Theta -1\right)\nu \notin Z$, the following two equalities hold true:*

(178) $$ℜG_{\Theta ,\nu }\left(0\right)=1$$

*and*

(179) $${\left(\frac{1}{\pi }{⨏}_{-\infty }^{+\infty }\frac{ℑG_{\Theta ,\nu }\left(\tau \right)}{\omega -\tau }d\tau \right)}_{\omega =0}=\frac{2}{\pi \nu }\mathrm{atan}\left(\tan(\frac{\pi }{2}\nu \Theta )\right)\neq 1.$$

*Because both functions are locally Hölder continuous, the K–K relations hold true in $L^{2}\left(R\right)$ iff they hold true pointwise. Hence, the transfer function $G_{\Theta ,\nu }\left(\omega \right)$ within the considered range of parameters is not causal.*

In the case of distributions, Theorem 11 can also be helpful. Let us first consider the following example:

**Example** **23.** *Let us return to the case of ν=3,5,7… mentioned in Example 1. We are going to show that the transform*

(180) $$G_{\nu }\left(\omega \right)=e^{-z\sqrt{\mu_{\gamma }\epsilon_{\beta }}{\left(j\omega \right)}^{\nu }}=\cos\left(z\sqrt{\mu_{\gamma }\epsilon_{\beta }}\omega^{\nu }\right)\pm j\sin\left(z\sqrt{\mu_{\gamma }\epsilon_{\beta }}\omega^{\nu }\right),$$

*where the sign depends on the parity of l∈N for ν=2l+1, is not causal. As one can see, the distribution*

(181) $$\frac{G_{\nu }\left(\omega \right)}{j\omega }=\pm \frac{\sin(z\sqrt{\mu_{\gamma }\epsilon_{\beta }}\omega^{\nu })}{\omega }-jp.v.\frac{\cos(z\sqrt{\mu_{\gamma }\epsilon_{\beta }}\omega^{\nu })}{\omega }$$

*belongs to $D_{L^{2}}^{\prime }$. Hence one can check if the distributional relation (89) is satisfied. Let us denote*

$$f\left(\omega \right)=ℜ\frac{G_{\nu }\left(\omega \right)}{j\omega }=\pm \frac{\sin(z\sqrt{\mu_{\gamma }\epsilon_{\beta }}\omega^{\nu })}{\omega }$$

*and*

$$g\left(\omega \right)=ℑ\frac{G_{\nu }\left(\omega \right)}{j\omega }=\;p.v.\frac{\cos(z\sqrt{\mu_{\gamma }\epsilon_{\beta }}\omega^{\nu })}{\omega }.$$

*In order to state that $G_{\nu }\left(\omega \right)$ is a causal transform, one needs to check that*

(182) $$G_{\nu }\left(\omega \right)=\omega \left(H(f)+jH(g)\right).$$

*As one can see, the function f(ω) satisfies the assumptions of the Wood Theorem 11. Moreover, it is an $L^{2}\left(R\right)$ function, so the distributional Hilbert transform can be replaced by a standard $L^{2}$ transform. By the Wood Theorem 11, the real part of the right-hand side of (182), i.e., $ℜG_{\nu }\left(\omega \right)$, is a locally Hölder function. It means that*

$$\cos\left(z\sqrt{\mu_{\gamma }\epsilon_{\beta }}\omega^{\nu }\right)=\pm \omega \left(H(f)(\omega )\right)$$

*for all ω∈R. This is obviously violated for ω=0, and it proves that (89) is not satisfied. Hence $G_{\nu }\left(\omega \right)$ is not causal.*

This example can be a good starting point for some general observations in the context of Theorem 4, providing an easily verifiable necessary condition for causality.

**Theorem** **12.** *Let us assume that $F={\left(j\omega \right)}^{k}G$, where $G\in D_{L^{q}}^{\prime }$, and that (89) holds, i.e.,*

$$F=\frac{{\left(j\omega \right)}^{k}}{j}H\left(G\right).$$

*Then, if the real (respectively imaginary) part of G is a locally Hölder continuous $L^{q}$ function, then its imaginary (respectively real) part must also be a locally Hölder continuous $L^{q}$ function.*

### 5.6. Causality Tests for Refractive Index

One can formulate the causality problem for media described by the refractive index n=n(ω)∈C. From the point of view of Maxwell’s equations, the velocity of electromagnetic wave is described by ϵ(ω) and μ(ω). Hence one obtains that $n^{2}\left(\omega \right)=c\mu \left(\omega \right)\epsilon \left(\omega \right)$, refer to (59). Let us assume that the dielectric-relaxation function $\chi_{e}\left(\omega \right)$ satisfies the assumptions of the Titchmarsh Theorem 3. Therefore, among others, it belongs to $L^{2}\left(R\right)$ and is causal. Then $\chi_{e}\left(\omega \right)$ has a holomorphic extension to the right half-plane, and the permittivity $\epsilon \left(\omega \right)=\epsilon_{0}\left(1+\chi_{e}\left(\omega \right)\right)$ also has a holomorphic extension to the complex right half-plane (the same considerations are applicable to the permeability $\mu \left(\omega \right)=\mu_{0}\left(1+\chi_{m}\left(\omega \right)\right)$). One should notice that this condition formulated for $e^{-i\omega t}$ settings implies that the considered function is holomorphic in the upper half-plane. We should mention that the existence of holomorphic extension is not a sufficient condition for causality, i.e., the behavior in *∞* is important as well. In general, assuming that $\chi_{e}\left(\omega \right)$ and $\chi_{m}\left(\omega \right)$ belong to $L^{2}\left(R\right)$ does not imply that the product $\chi_{e}\left(\omega \right)\chi_{m}\left(\omega \right)\in L^{2}\left(R\right)$. Hence, one has to be careful when deciding whether $n^{2}\left(\omega \right)-1$ is an $L^{2}\left(R\right)$ function. Similarly, if the holomorphic extensions ${\tilde{\chi }}_{e}\left(s\right)$ and ${\tilde{\chi }}_{m}\left(s\right)$ satisfy the assumptions (ii) of Theorem 3, it does not mean that these assumptions are satisfied by the product ${\tilde{\chi }}_{e}\left(s\right){\tilde{\chi }}_{m}\left(s\right)$. This means that we may not form conclusions about causality based only on the existence of holomorphic extension. Some assumptions concerning the behavior of the product ${\tilde{\chi }}_{e}\left(\sigma +j\omega \right)\cdot {\tilde{\chi }}_{m}\left(\sigma +j\omega \right)$ for a fixed σ>0 and |ω|→+∞ are needed as well. As proposed by Stockman [27], the K–K relations can be written for the complex refractive index as

(183) $$ℜn^{2}\left(\omega \right)-1=\frac{2}{\pi }{⨏}_{0}^{+\infty }\frac{\tau ℑn^{2}\left(\tau \right)}{\omega^{2}-\tau^{2}}d\tau$$

(184) $$ℑn^{2}\left(\omega \right)=-\frac{2\omega }{\pi }{⨏}_{0}^{+\infty }\frac{ℜn^{2}\left(\tau \right)-1}{\omega^{2}-\tau^{2}}d\tau .$$

Although the assumption $\chi_{e}\in L^{2}\left(R\right)$ does not imply that $\lim_{|\omega |\to +\infty }\chi_{e}\left(\omega \right)$ exists, in practical terms it is natural to assume that $\lim_{|\omega |\to +\infty }\chi_{e}\left(\omega \right)=0$. Due to this assumption regarding the electric susceptibility $\chi_{e}\left(\omega \right)$, as well as the magnetic one, one obtains that $n^{2}\left(\omega \right)\to 1$ when ω→+∞ [74]. The usual physical justification behind this assumption is that an incident, oscillating, electromagnetic field entering any medium stimulates the charges in that medium to oscillate (light-matter interaction). However, for very high frequencies of the incident field (ω→+∞) the charges of the medium cannot respond, because they have a finite mass, hence their inertia. As a result, for those very high frequencies, it is as if the field ‘sees’ a vacuum ($n^{2}\left(\omega \right)\to 1$), because it effectively does not interact with the medium at all.

From this additional assumption (i.e., $\lim_{|\omega |\to +\infty }\chi_{e}\left(\omega \right)=0$, $\lim_{|\omega |\to +\infty }\chi_{m}\left(\omega \right)=0$), as well as the assumption that both functions $\chi_{e}$ and $\chi_{m}$ are bounded (it happens, e.g., when they are continuous), one can conclude that the product $\chi_{e}\cdot \chi_{m}$ belongs to $L^{2}\left(R\right)$. In this case, one knows that $n^{2}\left(\omega \right)-1$ belongs to $L^{2}\left(R\right)$. It allows one to say that the relations (183) and (184) imply causality of the transform $n^{2}\left(\omega \right)-1$ by the classical Titchmarsh Theorem 3. From the formal perspective, the assumptions related to limits of the functions $\chi_{e}$, $\chi_{m}$ as |ω|→+∞ and their boundedness can be relaxed (e.g., by saying that one of these functions is any $L^{2}\left(R\right)$ function, and the other one is a bounded $L^{2}\left(R\right)$ function), but it is far less natural and does not allow to conclude that $n^{2}\left(\omega \right)\to 1$ when ω→+∞.

A separate discussion is needed when the susceptibilities $\chi_{e}\left(\omega \right)$ and $\chi_{m}\left(\omega \right)$ are not $L^{2}\left(R\right)$ functions, e.g., when they are tempered distributions. In this case, one has to be careful when defining the product $n^{2}\left(\omega \right)=c\left(1+\chi_{e}\left(\omega \right)\right)\left(1+\chi_{m}\left(\omega \right)\right)$, as the product of two distributions is not necessarily well-defined. One can surely define $n^{2}\left(\omega \right)$ as a tempered distribution when both $\chi_{e}\left(\omega \right)$ and $\chi_{m}\left(\omega \right)$ are represented by locally integrable functions, which are bounded as |ω|→+∞ by some polynomial. In this case, the K–K relations can be checked in the distributional sense with the use of Theorem 4, but it requires dividing the function $n^{2}\left(\omega \right)$ or $n^{2}\left(\omega \right)-1$ by an appropriate power of (jω). Let us also notice that the K–K relations for $n^{2}\left(\omega \right)$ and $n^{2}\left(\omega \right)-1$ can be different. In the case considered above, when $n^{2}\left(\omega \right)-1$ belongs to $L^{2}\left(R\right)$, there is no need to divide $n^{2}\left(\omega \right)-1$ by any positive power of (jω) (i.e., one may take k=0 in the Formula (87) or (88)). On the other hand, $n^{2}\left(\omega \right)$ is not the function in $D_{L^{2}}^{\prime }$, and in order to verify any of the Equations (87) or (88), one should take k=1.

In literature one can also find the K–K relations formulated for $n\left(\omega \right)=\sqrt{n^{2}\left(\omega \right)}$

(185) $$ℜn\left(\omega \right)-1=\frac{2}{\pi }{⨏}_{0}^{+\infty }\frac{\tau ℑn(\tau )}{\omega^{2}-\tau^{2}}d\tau$$

(186) $$ℑn\left(\omega \right)=-\frac{2\omega }{\pi }{⨏}_{0}^{+\infty }\frac{ℜn(\tau )-1}{\omega^{2}-\tau^{2}}d\tau .$$

However, as it can be noticed in [27], the function $n\left(s\right)=\sqrt{n^{2}\left(s\right)}$ can be not holomorphic in the right half-plane s∈C, even if $n^{2}\left(s\right)$ is holomorphic. For instance, when $n^{2}\left(s\right)$ approaches zero, then the derivative of its square root does not exist. It clearly shows that causality of n(ω) is not necessarily equivalent to the case of $n^{2}\left(\omega \right)$ causality. Apart from the problem with being holomorphic in $C_{+}$, there is also the problem of its behavior as |ω|→+∞. If $n\left(\omega \right)-1\in L^{2}\left(R\right)$, then it does not necessarily mean that $n^{2}\left(\omega \right)-1\in L^{2}\left(R\right)$. Moreover, if $n^{2}\left(\omega \right)-1\in L^{2}\left(R\right)$, then it is not necessarily true that $n\left(\omega \right)-1\in L^{2}\left(R\right)$. In order to draw any causality conclusions from the relations (185) and (186) formulated for n(ω), one should know that n(ω)−1 belongs to $L^{2}\left(R\right)$. Despite these issues, the K–K relation of the type (185) is successfully used for the interpretation of experimental results in [75]. That is, the effect of a femtosecond-laser-induced electronic band-gap shift on the refractive index is explicitly studied with the use of the K–K relations. Clearly, from this relation, a change in the absorption described by ℑn(ω) curve in turn affects ℜn(ω). It is worth mentioning that the K–K relations (185) and (186) formulated for n(ω) are valid for a single mode propagation in waveguides (e.g., optical), for which one can write $n\left(\omega \right)=k/k_{0}$ where $k_{0}=\omega /c$.

Let us now assume that $n\left(\omega \right)-1=\sqrt{n^{2}\left(\omega \right)}-1$ is causal (e.g., it is an $L^{2}\left(R\right)$ function which satisfies (185) and (186)). Then, if $N\left(t\right)=F^{-1}\left(n-1\right)$ is such a distribution that the convolution N∗N exists, and if it is a tempered distribution (the convolution of two causal distributions is causal as well), then ${\left(n\left(\omega \right)-1\right)}^{2}=F\left(N\right)\cdot F\left(N\right)=F\left(N*N\right)$ is also causal. However, the opposite theorem is false in general. Nevertheless, if one knows that the function $n^{2}\left(s\right)$ is holomorphic in $C_{+}$ and it does not achieve 0, then n(s) is holomorphic as well.

Still, one can prove that the passivity of media implies that $n\left(\omega \right)=\sqrt{n^{2}\left(\omega \right)}$ is holomorphic in the right half-plane [76]. The problem of choice between n(ω) and $n^{2}\left(\omega \right)$ for applications in optics is debated in [77]. That is, the consideration of propagation of optical pulses with the use of complex index of refraction is inconvenient in general. Therefore, when calculating the wave vector, one can take either $ℜ\sqrt{n^{2}\left(\omega \right)}$ or $\sqrt{ℜn^{2}\left(\omega \right)}$ as a velocity of wave propagation. Whereas the difference between the two is negligible for small losses, it is significant in other cases. The analysis of pulse propagation demonstrates that the use of $ℜ\sqrt{n^{2}\left(\omega \right)}$ results in a wave vector different than that actually exhibited by the propagating pulse. On the other hand, the definition $\sqrt{ℜn^{2}\left(\omega \right)}$ always correctly calculates the wave vector of pulse, hence it is preferred in optical investigations. Moreover, for negative refraction media, when the sign of permittivity or permeability changes as a frequency function, one should notice that the derivative of n(ω) does not exist when n(ω) approaches zero. One should take all these issues into consideration when either the K–K relations (183) and (184) or (185) and (186) are applied.

It is worth noticing that the K–K relations are also applicable—to a certain extent—in nonlinear optics. That is, the response function in the time domain should also be causal for nonlinear media. In general, nonlinear complex susceptibilities can have poles not only on a half of the complex-frequency plane; however, there are cases when it happens. In such a case, when holomorphic properties are available on a half of the complex-frequency plane, the standard K–K relations (83) and (84) can be useful [78]. The review of nonlinear K–K relations in optics and photonics is presented in [79], where it is shown that the nonlinear dispersion relations have a common form that can be understood in terms of the linear K–K relations (83) and (84) applied to a new electromagnetic system consisting of the material and the perturbation of its parameters. As noticed in [80], the nonlinear K–K relations are useful in optics and photonics to predict that an enhancement in the nonlinear optical absorption for a specific wavelength usually leads to a decrease in the nonlinear optical refraction associated with a considered material.

Equation (183) can be used to derive the condition of negative refraction with no (or low) loss at the observation frequency [27], i.e.,

(187) $$\frac{2}{\pi }\int_{0}^{+\infty }\frac{\epsilon^{\prime \prime }\left(\tau \right)\mu^{\prime }\left(\tau \right)+\mu^{\prime \prime }\left(\tau \right)\epsilon^{\prime }\left(\tau \right)}{{\left(\tau^{2}-\omega^{2}\right)}^{2}}\tau^{3}d\tau \leq -1.$$

It is derived assuming that, at and near the observation frequency ω, the material is transparent (e.g., the losses are compensated by gain), which mathematically implies that $ℑ\left[n^{2}\left(\omega \right)\right]=0$ and $\partial ℑ\left[n^{2}\left(\omega \right)\right]/\partial \omega =0$. Furthermore, it is assumed that the negative refractive index is implied by the condition $v_{p}\cdot v_{g}<0$, where $v_{p}$ and $v_{g}$ denote, respectively, the phase and group velocity. Due to these limitations, the condition (187) is replaced in [28] by the condition which does not require that $ℑ\left[n^{2}\left(\omega \right)\right]=0$ and $\partial ℑ\left[n^{2}\left(\omega \right)\right]/\partial \omega =0$ at the observation frequency. The condition (187), obtained from the K–K relations, implies that compensation of the optical losses or significant reduction, by any means (material or structural) of the imaginary part of permittivity and permeability, can also change the real parts of these quantities in such a way that the negative refraction disappears [27]. Concerning (183) and (187), care should be exercised for the case when both the real part of permittivity $\epsilon^{\prime }\left(\omega \right)$ and the real part of permeability $\mu^{\prime }\left(\omega \right)$ are negative–simultaneously and within the same frequency region, because in that case, although the product $\epsilon^{\prime }\left(\omega \right)\mu^{\prime }\left(\omega \right)$ is positive, one should nonetheless select the root with negative sign of the real part of $\sqrt{n^{2}\left(\omega \right)}$ and ensure that, in the absence of a gain mechanism the medium does remain passive [81]. Further, one can notice that (187) stipulates that it is impossible to have a loss-free or amplifying ($\epsilon^{\prime \prime }\left(\omega \right)\leq 0$, $\mu^{\prime \prime }\left(\omega \right)\leq 0$) medium with a negative refractive index ($\epsilon^{\prime }\left(\omega \right)<0$, $\mu^{\prime }\left(\omega \right)<0$, $n^{\prime }\left(\omega \right)=ℜn(\omega )<0$) for all the frequencies–or else (187) would not hold true. However, (187) does not preclude the possibility that such a medium exists within a finite-bandwidth frequency region, outside which the product in the numerator in the integral of (187) could make a sufficiently ‘negative’ contribution, so that the overall (187) still holds true. Indeed, such lossless negative-refractive-index media have been reported in the past, both experimentally [82] and numerically [83,84,85].

### 5.7. Summary

The described analytical methods for causality evaluation of dielectric models of photonic materials are summarised in Table 1.

## 6. Conclusions

In this article, a comprehensive analysis of mathematical techniques for causality evaluation of photonic materials is presented. It includes not only the approaches valid for the $L^{2}$ functions, i.e., those for which the Titchmarsh theorem can be useful, but also the functions to which the distribution theory and the FO calculus have to be applied. We present a set of theorems applicable for causality evaluations, as well as specific examples showing how to use this mathematical machinery. Furthermore, the set of various distributional theorems presented in literature is collected as the distributional version of the Titchmarsh theorem, allowing us to evaluate causality of complicated electromagnetic systems on a mathematically rigorous basis. In addition to the well-known K–K relations, we have also outlined four further methodologies, namely application of the Paley–Wiener theorem, calculation of the inverse Fourier transformation, identification of holomorphic extensions to the right half-plane, and check of the K–K relations for the natural logarithm of a system’s frequency response. The collection of these methodologies—otherwise scattered in a wide range of pertinent literature—may prove useful for scientists and engineers investigating causality problems in electrodynamics and optics.

## Figures and Tables

**Table 1:** Summary of causality evaluation methods for photonic materials (IFT—inverse Fourier transformation, KKR—K–K relations, HE—holomorfic extension, PWT—Paley-Wiener theorem).

Model	Equation	Method	Example
dielectric with constant permittivity	(35)	IFT	2
dielectric with ohmic losses	(38)	IFT	3
Djordjevic-Sarkar for lossy dielectric [6,41]	(41)	IFT	9
Westerlund [42,43]	(42)	KKR	17
power-law for porous media [45]	(44)	KKR	17
generalized power-law [25]	(65) and (66)	PWT	1
Debye relaxation [46,47]	(45)	IFT	4
Lorentz [7,47]	(46)	IFT	5
Lorentz in high-frequency limit [7]	(47)	IFT, KKR	6, 20
Lorentz with static magnetic induction [7]	(48)	IFT	7
Lorentz in FO generalization [49]	(49)	HE	14
Drude [47]	(50)	IFT	8
Cole-Cole [46,50,51,52]	(51)	HE	10
Cole-Davidson [46,53]	(52)	HE	11
Havriliak-Negami [46,54,55]	(53)	HE	12
Raicu [46,56]	(54)	HE	13
